# Vision toolkit part 1. Neurophysiological foundations and experimental paradigms in eye-tracking research: a review

**DOI:** 10.3389/fphys.2025.1571534

**Published:** 2025-06-19

**Authors:** Quentin Laborde, Axel Roques, Matthieu P. Robert, Allan Armougum, Nicolas Vayatis, Ioannis Bargiotas, Laurent Oudre, Pierre-Paul Vidal

**Affiliations:** ^1^ Université Paris Saclay, Université Paris Cité, ENS Paris Saclay, CNRS, SSA, INSERM, Centre Borelli, Gif-sur-Yvette, France; ^2^ SNCF, Technologies Department, Innovation and Research, Saint Denis, France; ^3^ Thales AVS France, Training and Simulation, Osny, France; ^4^ Ophthalmology Department, Hôpital Universitaire Necker-Enfants malades, AP-HP, Paris, France; ^5^ Université Paris-Saclay, Inria, CIAMS, Gif-sur-Yvette, France; ^6^ Institute of Information and Control, Hangzhou Dianzi University, Hangzhou, Zhejiang, China

**Keywords:** eye-tracking, experimental settings, review article, human gaze behavior, oculomotor control

## Abstract

Eye-tracking research offers valuable insights into human gaze behavior by examining the neurophysiological mechanisms that govern eye movements and their dynamic interactions with external stimuli. This review explores the foundational principles of oculomotor control, emphasizing the neural subsystems responsible for gaze stabilization and orientation. Although controlled laboratory studies have significantly advanced our understanding of these mechanisms, their ecological validity remains a critical limitation. However, the emergence of mobile eye tracking technologies has enabled research in naturalistic environments, uncovering the intricate interplay between gaze behavior and inputs from the head, trunk, and sensory systems. Furthermore, rapid technological advancements have broadened the application of eye-tracking across neuroscience, psychology, and related disciplines, resulting in methodological fragmentation that complicates the integration of findings across fields. In response to these challenges, this review underscores the distinctions between head-restrained and naturalistic conditions, emphasizing the importance of bridging neurophysiological insights with experimental paradigms. By addressing these complexities, this work seeks to elucidate the diverse methodologies employed for recording eye movements, providing critical guidance to mitigate potential pitfalls in the selection and design of experimental paradigms.

## 1 Introduction

Eye movements are controlled by three pairs of agonist-antagonist extra-ocular muscles. The lateral and medial recti generate horizontal movements, while the superior rectus, inferior rectus, superior oblique, and inferior oblique—collectively known as cyclovertical muscles—work together to produce both vertical and torsional rotations (Leigh and Zee, 2015). Sherrington’s law of reciprocal innervation states that the contraction of an ocular muscle is paired with the inhibition of its antagonist, while Hering’s law of equal innervation ensures equal neural input to synergistic muscles in both eyes for coordinated movements (Allary, 2018). Human oculomotor control is generally assumed to be governed by five distinct neural subsystems: the vestibulo-ocular reflex (VOR), the optokinetic reflex (OKR), the saccadic system, the smooth pursuit system, and the vergence system (Robinson et al., 1981; Büttner and Büttner-Ennever, 1988; Duchowski and Duchowski, 2017).

Early research on the neural pathways governing motor neurons of the extraocular muscles led researchers to adopt reductionist approaches in controlled laboratory environments, which limited natural human behavior. However, studies suggest that these artificial constraints, particularly the restriction of head movements, can alter the true functioning of the oculomotor system, leading to biased representations of its behavior in natural contexts (Dorr et al., 2010). This bias arises not only from isolating neural subsystems with controlled stimuli, but also from the recording methods themselves, which require stabilizing the head with devices such as chin rests and chin bars (Eggert, 2007; Wade, 2010; Wade, 2015).

More recently, the increasing availability of mobile eye trackers has significantly advanced the study of eye movements in natural, or ecological, settings (Kothari et al., 2020). However, analyzing eye movement data in such conditions remains challenging. Natural gaze exploration involves simultaneous movements of the eyes, head, trunk, and feet, and it has been shown that the properties of eye movements recorded in these contexts differ from those in laboratory settings (Carnahan and Marteniuk, 1991; Land, 1992; Land, 2004; Klein and Ettinger 2019). While lab experiments typically target specific subsystems, natural eye movements result from the combined action of multiple neural pathways. Indeed, automatic reflexes like the VOR and the OKR, as well as higher-order cognitive processes, motor signals, and sensory inputs, all contribute to ocular motoneuron activity during body movement (Anastasopoulos et al., 2009). The complexity of eye movement physiology and the limited studies available hinder a full understanding of eye movements in ecological contexts.

On the other hand, the growing accessibility of eye movement recording technologies has led to their integration across various research fields, such as neuroscience, marketing, psychology, and medicine, fostering the development of specialized communities. Each discipline has contributed significantly to advancing eye movement research. However, this rapid growth has also caused fragmentation, with insights dispersed across a wide range of literature. Since each field often pursues distinct goals, methodologies and findings are typically field-specific, limiting their applicability across disciplines. This review examines the neurophysiology of eye movements and the experimental paradigms employed in this field, with the aim of synthesizing studies of the oculomotor system across different research communities. Given the extensive scope of the topic, this review is not intended to be exhaustive; rather, it highlights key physiological insights into gaze control mechanisms. The objective is to inform the design of experimental protocols for investigating eye movements, both in controlled environments and in more ecologically valid settings—particularly those without physical constraints on head movements. It is important to note that this brief review focuses solely on the characteristics and description of ocular movements and does not explicitly address visual behavior or the allocation of visual attention.

With a primary emphasis on findings from human studies and on the functional aspects of eye movement, the following sections offer an overview of current knowledge on major eye movement types. This work distinguishes findings obtained under controlled laboratory conditions 2 — from those derived in more natural, head-free environments—see Section 3. Finally, building on these neurophysiological insights, we discuss practical considerations to support researchers in designing experimental protocols in Section 4.

This review stands at the intersection of multiple contributions in the existing literature, providing an overview of eye movements with a clear distinction between findings obtained under controlled laboratory conditions and those from ecological contexts. While aligned with prior works such as Lappi (2016), it is less exhaustive than the comprehensive treatment in Leigh and Zee (2015), which delves into the neurophysiology, neural circuits, and models underlying saccadic and smooth pursuit movements. Our goal is to offer foundational knowledge for researchers interested in integrating eye-tracking methodologies into their studies. The practical section of this review—highlighting methods for recording and analyzing eye-tracking data—distinguishes it from more theoretical works, aligning more closely with reviews focused on practical considerations (Singh and Singh, 2012; Lim et al., 2020; Klaib et al., 2021) or best practices in data acquisition (Carter and Luke, 2020). In summary, this work provides a concise synthesis of key knowledge on the neurophysiology of eye movements with a practical focus. By bridging theoretical insights and practical applications, it aims to help researchers develop robust experimental protocols.

## 2 When the head is physically restrained

Most laboratory protocols for studying eye movements are performed with the head constrained. In these conditions, gaze reorientation relies exclusively on eye movements. The following sections outline the canonical components of eye movements under such laboratory settings, *i.e.*, saccades, smooth pursuits, fixational eye movements as well as the vestibulo-ocular reflex (VOR) and optokinetic reflex (OKR).

### 2.1 Saccades

Saccades are rapid, ballistic eye movements that typically occur at a frequency of 2−4 times per second, comprising approximately 10% of total viewing time (Noton and Stark, 1971; Klein and Ettinger, 2019). However, this frequency can vary considerably depending on perceptual and cognitive demands (Fischer and Weber, 1993a). The amplitude of saccadic movements generally reaches a maximum of 40−−45 degrees, which is somewhat less than the oculomotor range of around 53° (Freedman, 2008). Following each saccade, a refractory period of approximately 120−200 milliseconds is commonly observed (Robinson, 1968; Zuber et al., 1968), though Robinson also documented cases where successive saccades occurred within shorter intervals (Robinson, 1968). Fischer and Boch’s pioneering studies in monkeys (Fischer and Boch, 1983) revealed a bimodal distribution of saccadic latency, distinguishing two populations: one with short latencies of 80−−120 milliseconds, termed *express saccades*, and another with latencies of 120−−200 milliseconds, termed *fast regular saccades*. Similar latency patterns have been observed in humans, though the prevalence of distinct express and regular saccade populations varies among individuals (Wenban-Smith and Findlay, 1991; Kingstone and Klein, 1993). Express saccades are more likely to occur with predictable targets and following extensive training.

Functionally, saccades can be categorized as either reflexive, also known as *visually guided* (Klein and Ettinger, 2019), or *volitional* in nature (Pierrot-Deseilligny et al., 1995; Patel et al., 2012; Leigh and Zee, 2015). These two saccade types are controlled by parallel subsystems (Patel et al., 2012): visually guided saccades are primarily driven by external stimuli, while volitional saccades are internally generated, relying more on cognitive processes like attention, inhibition, and working memory (Seideman et al., 2018; Klein and Ettinger, 2019). Volitional saccades include tasks like predictive saccades, where eye movements anticipate a target’s appearance based on learned temporal or spatial patterns, such as tracking a stimulus appearing rhythmically at predictable locations (Leigh and Zee, 2015), and memory-guided saccades, which direct gaze toward a remembered target location without current visual input, engaging working memory to recall the target’s position (Seideman et al., 2018). Similarly, antisaccades require suppressing a reflexive saccade toward a sudden stimulus to instead look at the opposite location, relying on inhibitory control and attention as a measure of cognitive flexibility (Klein and Ettinger, 2019), while saccade sequencing involves planning and executing a series of saccades in a specific order to multiple targets, integrating attention, working memory, and motor planning for precise coordination (Patel et al., 2012).

These tasks highlight the cognitive demands of volitional saccades, distinguishing them from reflexive saccades while illustrating their interplay along a continuum of saccadic control (Klein and Ettinger, 2019). Earlier work hinted at these mechanisms, with Bahill et al. (1981) observing that intrinsic saccade properties—such as peak velocity, amplitude, and duration—were influenced by higher-order cognitive factors like attention, muscle fatigue, and tiredness. Importantly, the separation between reflexive and volitional saccades should be understood as a continuum rather than a strict dichotomy, as internal cognitive motivations and decision-making processes influence both saccade types (Klein and Ettinger, 2019).

Saccadic eye movements are generated by a distributed network of cortical and subcortical structures. The frontal eye fields (FEF), supplementary eye fields (SEF), and posterior parietal cortex (PPC) initiate voluntary and goal-directed saccades by sending commands to the superior colliculus (SC) and brainstem saccade generators (Leigh and Zee, 2015; Pierrot-Deseilligny et al., 2004). The SC, particularly its intermediate and deep layers, integrates multisensory inputs and contributes to both reflexive and voluntary saccades (Wurtz and Goldberg, 1972). Premotor structures in the brainstem, including the paramedian pontine reticular formation (PPRF) for horizontal saccades and the rostral interstitial nucleus of the medial longitudinal fasciculus (riMLF) for vertical and torsional saccades, generate high-frequency burst activity. These work in conjunction with omnipause neurons in the nucleus raphe interpositus, which inhibit saccade initiation and regulate timing (Scudder et al., 2002). The cerebellum, especially the fastigial nucleus and dorsal vermis, refines saccadic metrics and mediates motor learning and adaptation (Optican and Robinson, 1980; Robinson and Fuchs, 2001). This network operates both hierarchically and in parallel, integrating sensory, cognitive, and motor information to guide rapid eye movements.

Saccade kinematics are typically characterized by a stereotyped, symmetrical velocity profile for movements ranging from 5 to 25°, with larger saccades tending to display a skewed profile, where the deceleration phase is often longer than the acceleration phase. Saccades also exhibit a linear duration-amplitude relationship, with the slope estimated to be between 1.5 and 3 milliseconds per degree, as well as a non-linear relationship between peak velocity and amplitude (Bahill et al., 1981; Klein and Ettinger, 2019). This latter relationship is commonly referred to as the *main sequence*, a term introduced by Bahill et al. (1975) and borrowed from astronomy, which has since become a major focus of research (Freedman, 2008; Gibaldi and Sabatini, 2021). Notably, it has been observed that the peak velocity of a saccade increases as a function of its amplitude, reaching a peak at approximately 20−−30 degrees (Bahill et al., 1975; Zuber et al., 1968), after which it plateaus around 600° per second. Alternative models for the main sequence have been proposed in subsequent studies (Leigh and Zee, 2015; Gibaldi and Sabatini, 2021). Nonetheless, the main sequence remains a valuable tool for studying both pathological and non-pathological eye movements in clinical neuroscience (Leigh and Kennard, 2004; Ramat et al., 2006), for developing and evaluating neural models of saccadic eye movement control (Becker, 1989; Robinson et al., 1993; Quaia et al., 1999; Jagadisan and Gandhi, 2017), and for investigating eye movement adaptation (Optican and Robinson, 1980).

At the end of a typical saccadic eye movement, just before settling into steady fixation, the pupil signal often shows a damped oscillation, with one or two observable cycles before attenuation (Nyström et al., 2013b; Hooge et al., 2015). These *post-saccadic oscillations* (PSOs) typically have an amplitude of around 2°, with oscillation periods averaging about 20 milliseconds (Hooge et al., 2015). The origin of PSOs, long debated, is now believed to be due to dynamic deformations of the iris’s inner edge during saccades (Nyström et al., 2013b; Hooge et al., 2016). Specifically, these oscillations result from movements of the pupil within the eyeball, referred to as *iris wobbling* or the *eye wobbling phenomenon*. It’s important to note that PSO characteristics can vary significantly depending on the eye-tracking methods used (Hooge et al., 2016), the direction of the saccade (Hooge et al., 2015), and individual differences, such as the observer’s age (Mardanbegi et al., 2018) and pupil size (Nyström et al., 2016).

Saccade metrics were found to be stable within and across trials, thereby making them suitable biometric data for authentication, identification or to reveal differences in perceptual-motor style between individuals (Klein and Ettinger, 2019; Vidal and Lacquaniti, 2021). For example, the pioneering work of Holland and Komogortsev (2013) and Rigas and Komogortsev (2016) demonstrated the robustness of individual-specific eye movements characteristics for recognition purposes with different types of visual stimulus. Their approach led to the development of the *complex eye movement extended* biometrics, which consists of several fixation and saccade-related characteristics that together constitute an individual’s biometric fingerprint. While these approaches do not yet represent a realistic alternative to existing biometric standards, they represent a promising field of research.

In neurological and psychiatric disorders, abnormalities in saccadic eye movements provide insights into impaired motor planning, inhibitory control, and neural circuit dysfunction. In Parkinson’s disease (PD), saccades typically exhibit hypometria—reduced amplitude—and prolonged latencies, particularly for volitional saccades. These deficits stem from dysfunction in the basal ganglia, supplementary eye fields, and frontal eye fields (FEF), which impair the generation and execution of planned movements (Terao et al., 2011; Lal and Truong, 2019). Huntington’s disease (HD) is associated with increased antisaccade latencies and high error rates, reflecting early degeneration of the striatum and prefrontal cortex, both critical for suppressing automatic responses. Antisaccade errors in HD may precede overt motor symptoms and serve as early markers of cognitive decline (Lal and Truong, 2019). In progressive supranuclear palsy (PSP), vertical saccades—particularly downward—are severely impaired due to degeneration of the rostral interstitial nucleus of the medial longitudinal fasciculus (riMLF) and midbrain structures (Leigh and Zee, 2015). Cerebellar disorders, such as spinocerebellar ataxias, result in dysmetric saccades—overshooting—hypermetria—or undershooting—hypometria—of the target—and poor saccadic adaptation. These effects are attributed to damage in the dorsal vermis and fastigial nucleus, which modulate saccadic accuracy.

In schizophrenia, antisaccade errors are markedly increased and latencies highly variable, indicating core deficits in inhibitory control and executive functioning. These deficits are linked to dysfunction in the dorsolateral prefrontal cortex (DLPFC) and its connections with the FEF and basal ganglia. Impaired antisaccade performance is considered a potential endophenotype for schizophrenia (Gooding and Basso, 2008). Similarly, individuals with attention-deficit/hyperactivity disorder (ADHD) demonstrate increased antisaccade error rates and variable reaction times, pointing to immature or dysfunctional prefrontal inhibitory mechanisms (Munoz et al., 2003). These deficits reflect challenges in voluntary response suppression and sustained attention. Lastly, saccadic intrusions, such as square-wave jerks—involuntary saccades that briefly displace fixation—are common across neurodegenerative disorders and may interfere with steady gaze. While not volitional, these intrusions further signal brainstem or cerebellar dysfunction (Leigh and Kennard, 2004).

### 2.2 Smooth pursuit

Ocular pursuit movements are triggered primarily by the continuous motion of a target, causing its image to drift across the retinal surface, and their primary function is to preserve visual acuity by stabilizing the moving image on or near the fovea. The primary input driving these movements is the retinal slip velocity, which refers to the relative motion of the target across the retina (Binder et al., 2009; Klein and Ettinger, 2019). In contrast, saccadic eye movements are typically triggered by discrete positional changes, such as when a target suddenly jumps outside the foveal region, to rapidly recenter the target’s image on the fovea. Unlike the saccadic system, which operates in discrete bursts, the smooth pursuit system is continuous and does not exhibit a refractory period (Robinson, 1965). Typical optimal pursuit speeds range from 15 to 30° per second (Rashbass, 1961; Meyer et al., 1985; Ettinger et al., 2003; Klein and Ettinger, 2019), although efficient tracking of velocities up to 100° per second has been observed for predictable motion patterns. This suggests that pursuit control involves higher-level extra-retinal mechanisms, such as anticipation and predictive processes.

Smooth pursuit movements consist of two phases. The initial phase, known as *pursuit initiation*, is driven solely by visual motion information. It is characterized by a latency period—the time required for the eyes to begin tracking the target after it starts moving—which ranges between 120 and 180 milliseconds in healthy individuals, depending on task conditions and experience (Klein and Ettinger, 2019). During the first 100 milliseconds of pursuit initiation, the response is based solely on the initial appearance of the target, unaffected by changes in the retinal image due to eye movement. In this phase, pursuit operates in an *open-loop* manner, relying on target movement without feedback from eye position. This *open-loop* phase can be modified by experience as the system adapts to changes in target velocity, a process known as *pursuit adaptation* (Chou and Lisberger, 2004).

The second phase, *pursuit maintenance*, aims to stabilize the target on the fovea. It combines visual feedback with predictions of target velocity to maintain the image within the zone of optimal visual acuity. In this *closed-loop* phase, any deviations from the ideal trajectory are corrected through compensatory eye movements (Thier and Ilg, 2005). Retinal velocity, image acceleration (Lisberger et al., 1987), and target position relative to the fovea (Blohm et al., 2005) all serve as error signals guiding pursuit. While pursuit is largely feedback-driven, cognitive factors like experience with target motion and stimulus predictability can modulate its performance (Barnes, 2008).

Smooth pursuit eye movements are controlled by an interconnected network of cortical, subcortical, brainstem, and cerebellar structures. The frontal eye fields (FEF), particularly their pursuit-related subregion, initiate and sustain voluntary tracking, while the lateral intraparietal area (LIP) modulates attentional focus and target selection (Tanaka and Lisberger, 2002; Thier and Ilg, 2005). Visual motion signals are primarily processed in the middle temporal (MT) and medial superior temporal (MST) areas, which compute retinal slip velocity and convey motion-related input to pursuit pathways (Newsome et al., 1985). These signals are relayed to the dorsolateral pontine nuclei (DLPN) in the brainstem, which project to the cerebellum to help generate smooth pursuit commands (Mustari et al., 1988). The cerebellum, especially the flocculus and posterior vermis, refines pursuit accuracy and supports adaptation through motor learning mechanisms (Miles and Fuller, 1975; Thier and Ilg, 2005).

Due to delays in the visual pathways and the limitations of eye velocity and acceleration, smooth pursuits are often supplemented by corrective or *catch-up* saccades. These rapid saccades are important for maintaining target tracking when smooth pursuit alone cannot compensate for unpredictable target movement or rapidly varying velocities, leading to retinal error accumulation (Haller et al., 2008). Catch-up saccades are highly controlled and executed without visual feedback, with their precision essential for effective pursuit. Research has shown that their amplitudes are closely aligned with both positional error and retinal slip (De Brouwer et al., 2002). For a comprehensive discussion of saccade-pursuit interactions, see the recent review by Goettker and Gegenfurtner (2021).

Interestingly, studies have demonstrated that the horizontal component of pursuit eye movements is more accurate than the vertical component (Rottach et al., 1996; Grönqvist et al., 2006; Ingster-Moati et al., 2009; Ke et al., 2013). This increased accuracy in horizontal tracking has been observed not only for targets moving strictly along the horizontal or vertical axes but also for horizontal and vertical components in bidirectional pursuit sequences (Ke et al., 2013). Moreover, horizontal pursuit mechanisms are found to develop earlier in children, supporting a developmental asymmetry in pursuit capabilities (Grönqvist et al., 2006). These directional differences align with findings indicating distinct neurophysiological substrates for horizontal and vertical pursuit pathways (Saito and Sugimura, 2020; Kettner et al., 1996; Chubb et al., 1984). The distinct neurophysiological substrates for horizontal and vertical pursuit pathways suggest independent feedback control mechanisms. For instance, Rottach et al. (1996) demonstrated that horizontal smooth pursuit in healthy subjects is more accurate and exhibits lower variability than vertical pursuit, with these differences persisting across horizontal, vertical, and diagonal target trajectories. This asymmetry is further supported by Rottach et al. (1997), who studied Niemann-Pick type C disease and found that horizontal and vertical saccades are independently affected, implying separate neural feedback loops for each axis. These findings suggest that horizontal pursuit relies on more robust control circuits, potentially involving the medial superior temporal area and pontine nuclei, while vertical pursuit engages distinct brainstem and cerebellar pathways, which may be less precise or more susceptible to disruption (Saito and Sugimura, 2020; Kettner et al., 1996). Such independent control underscores the functional and developmental differences observed in pursuit performance.

Aberrant smooth pursuit eye movements, characterized by impaired tracking of a moving target, serve as sensitive biomarkers for neurological and psychiatric disorders. In schizophrenia, reduced pursuit gain—eye velocity divided by target velocity—and increased phase lag reflect impaired motion processing in the middle temporal and medial superior temporal areas (MT/MST) and disrupted prefrontal control, particularly in the dorsolateral prefrontal cortex (DLPFC) (Chen et al., 1999; O’Driscoll and Callahan, 2008; Lencer et al., 2015). Cerebellar ataxias, such as spinocerebellar ataxia type 3 (SCA3), exhibit low-gain pursuit, irregular tracking, and frequent catch-up saccades, stemming from floccular and posterior vermal dysfunction that impairs motor learning and predictive pursuit (Miles and Fuller, 1975; Buttner et al., 1998). In Parkinson’s disease, pursuit gain is mildly reduced, especially for unpredictable target trajectories, due to basal ganglia deficits disrupting movement initiation and predictive control (Lekwuwa et al., 1999; Frei, 2021). Attention-deficit/hyperactivity disorder (ADHD) patients show fluctuating pursuit gain and elevated velocity errors, linked to frontoparietal attentional control impairments (Karatekin, 2007). Unlike saccadic disorders, which produce discrete spatial errors—*e.g.*, hypometria, square-wave jerks—pursuit dysfunction manifests as continuous tracking inaccuracies, notably altered gain and phase delay, quantifiable via high-resolution eye-tracking (Thier and Ilg, 2005).

### 2.3 Fixational eye movements

A fixation is defined as a period during which gaze is directed at a specific location, projecting the image onto the high-resolution processing region of the retina, the *fovea centralis*. Despite efforts to maintain a steady gaze, the eyes exhibit continuous, involuntary motion, influencing much of our visual experience. This creates a contradiction in the visual system: while gaze remains fixed on an object, the eyes are never entirely still. The precise roles of fixational eye movements—namely, *tremors*, *drifts*, and *microsaccades* (Martinez-Conde et al., 2004; Martinez-Conde, 2006) — in the visual process remain unclear and are the subject of ongoing discussion. It is believed that one function of these movements is to counteract neural adaptation by introducing small, random displacements of the retinal image. This helps ensure continuous stimulation of different photoreceptor cells in the fovea, preventing perceptual fading that would occur if the retinal image remained stationary (Pritchard, 1961). Additionally, fixational eye movements are proposed to play a role in the acquisition and processing of visual information by optimizing retinal sampling and enhancing the fine details of the visual scene (Klein and Ettinger, 2019).

#### 2.3.1 Tremors

Ocular micro-tremors, sometimes called physiological nystagmus, are tiny, high-frequency, involuntary eye oscillations that occur naturally in healthy eyes. These movements typically vibrate at 70–100 cycles per second—though some studies report a broader range of 50–200 cycles per second—with amplitudes smaller than 0.01° (Martinez-Conde et al., 2004; Collewijn and Kowler, 2008; Klein and Ettinger, 2019). As a normal feature of vision, micro-tremors are not a sign of disease but one of three types of fixational eye movements, alongside slow drifts and microsaccades, which together maintain clear vision during steady gaze. They originate from the rapid, asynchronous firing of fast-twitch motor units in the extraocular muscles, controlled by motor neurons in the brainstem’s motor nuclei (Ezenman et al., 1985; Collewijn and Kowler, 2008).

The neuroanatomy of micro-tremors centers on the brainstem’s extraocular motor nuclei—abducens, oculomotor, and trochlear—which send precise signals to the six extraocular muscles that position the eyes (Leigh and Zee, 2015). These nuclei produce high-frequency firing patterns that create the microscopic oscillations observed in micro-tremors (Ezenman et al., 1985). The pontine reticular formation, a brainstem region involved in coordinating gaze, likely refines the timing of these signals, contributing to the tremors’ rapid frequency (Sparks, 2002). Often described as neural *“noise”* in the ocular motor system, micro-tremors may serve a functional role. One hypothesis suggests they facilitate stochastic resonance, where subtle noise enhances the detection of faint visual signals, such as slight environmental shifts (Simonotto et al., 1997; Hennig et al., 2002). This idea remains speculative, however, and further research is needed to confirm its significance in visual processing.

Early research proposed that micro-tremors in each eye were independent (Riggs and Ratliff, 1951; Ditchburn and Ginsborg, 1953). More recent studies, however, have observed partial synchronization, evidenced by peaks in spectral coherence between the two eyes, likely mediated by shared neural pathways like the medial longitudinal fasciculus (Spauschus et al., 1999). This brainstem structure connects the abducens and oculomotor nuclei, enabling coordinated eye movements. The mechanisms driving this synchronization are not fully understood, highlighting an active area of investigation.

Studying micro-tremors is challenging because their high frequency often falls below the noise threshold of standard eye-tracking systems and can overlap with other eye movements, such as drifts or microsaccades (Klein and Ettinger, 2019). Despite these difficulties, advancements in high-precision technologies, including video-based systems, scleral search coils, and specialized devices, have enabled accurate measurements, confirming the tremors’ small amplitude and rapid frequency (Collewijn and Kowler, 2008; McCamy et al., 2013; McCamy et al., 2014). These movements contribute to retinal image stability, preventing visual fading—known as *Troxler fading*—during fixation (Engbert and Kliegl, 2004). By introducing subtle motion across the retina, micro-tremors may refresh visual input, supporting sharp, high-resolution vision and potentially aiding tasks requiring fine visual detail.

#### 2.3.2 Microsaccades

Microsaccades are small-amplitude saccadic eye movements, occurring approximately once or twice per second (Rolfs, 2009). While traditionally considered a type of fixational eye movement, emerging research suggests that microsaccades share neural pathways with larger saccades (Hafed, 2011) and exhibit many similar characteristics (Abadi and Gowen, 2004; Otero-Millan et al., 2013), notably adhering to the main sequence (Zuber et al., 1965). As such, microsaccades may be viewed as part of the broader continuum of saccadic movements. Interestingly, microsaccades are often regarded as involuntary or unconscious, yet they are regulated by the same endogenous control mechanisms that govern larger saccades (Collewijn and Kowler, 2008). Furthermore, assumption that humans are unaware of their microsaccades requires reconsideration, as individuals can exert a degree of control over them with appropriate training. For example, studies have demonstrated that individuals with experience in laboratory fixation tasks are capable of suppressing their microsaccades for several seconds during tasks requiring high visual acuity (Bridgeman and Palca, 1980; Steinman et al., 1967; Winterson and Collewijn, 1976).

The neuroanatomy underlying microsaccades involves a distributed network of brain regions that overlaps significantly with the neural circuitry responsible for larger saccadic eye movements. Key structures include the superior colliculus, which integrates sensory and motor signals to initiate microsaccades (Hafed, 2011), and the frontal eye fields, which contribute to their modulation, particularly in voluntary contexts (Tian et al., 2016). The brainstem, particularly the pontine reticular formation and the oculomotor nuclei, plays a critical role in generating the precise motor commands for these rapid eye movements (Scudder et al., 2002). Additionally, the cerebellum fine-tunes microsaccade amplitude and timing, ensuring their accuracy during fixation tasks (Otero-Millan et al., 2011). Neuroimaging and electrophysiological studies suggest that the same cortical and subcortical pathways that govern saccades are recruited for microsaccades, supporting the view that they are part of a continuum of oculomotor behavior (Martinez-Conde et al., 2009). This shared neural substrate enables the endogenous modulation of microsaccades, as seen in trained individuals who can suppress them to enhance visual acuity in specific tasks (Steinman et al., 1973).

Several studies have examined how anticipation affects microsaccade frequency. Betta and Turatto (2006) demonstrated that anticipating a motor response could reduce the microsaccade rate, while uncertainty about the motor response did not have the same effect (Rolfs, 2009). Similarly, anticipatory responses to sensory events can lead to a phenomenon called *oculomotor freezing*, characterized by a transient reduction in spontaneous microsaccade frequency lasting 100–400 milliseconds after the onset of an auditory, tactile, or visual stimulus.

The functional role of microsaccades remains a highly debated issue in the literature. Cornsweet (1956), Krauskopf et al. (1960) hypothesized that microsaccades help counteract the random drift of the eyes, serving a corrective role in both fixation position and binocular disparity—the slight difference between the retinal images of the left and right eyes. Other studies suggested that microsaccades may mitigate retinal adaptation by maintaining motion on the retina with respect to the visual environment (Ditchburn and Ginsborg, 1952; Riggs et al., 1953). Additional research suggests that microsaccades prevent retinal adaptation by promoting super-diffusive dynamics of gaze—where the gaze trajectory during fixation spreads faster than a normal random walk—over short time scales. Over longer time scales, the sub-diffusive dynamics of gaze—characterized by a slower spread of gaze trajectories compared to a normal random walk—mitigate fixation errors and reduce binocular disparity more effectively than an uncorrelated random walk (Engbert and Kliegl, 2004; Moshel et al., 2008; Roberts et al., 2013). Finally, some authors remain skeptical of the idea that microsaccades serve a unique role in sustaining fixation or preventing retinal adaptation, suggesting that these functions could be adequately fulfilled by smooth pursuit or slow drift movements (Collewijn and Kowler, 2008; Kowler, 2011; Klein and Ettinger, 2019). In fact, some researchers have even suggested that microsaccades represent an evolutionary enigma (Kowler and Steinman, 1980; Martinez-Conde et al., 2004).

Part of the confusion surrounding the functional role of microsaccades stems from ambiguity in their definition. Traditionally, microsaccades are distinguished from regular saccades by amplitude thresholds, with movements below a certain threshold classified as microsaccades. Early studies defined microsaccades as movements ranging from approximately 0.20–0.25° (Boyce, 1967; Cunitz and Steinman, 1969; Ditchburn and Foley-Fisher, 1967). Recent studies, however, have expanded the threshold to include movements up to 1−2 degrees (Engbert and Kliegl, 2004; Martinez-Conde et al., 2006). This broader range complicates direct comparisons with earlier literature and raises concerns regarding the functional interpretation of microsaccades.

#### 2.3.3 Drifts

Ocular drifts are slow, continuous eye movements occurring during inter-saccadic intervals, producing gaze trajectories that approximate a random walk—small, stochastic displacements with varying directions and amplitudes, typically shifting the retinal image by approximately 0.13° at velocities below 0.5° per second (Cornsweet, 1956; Engbert and Kliegl, 2004; Collewijn and Kowler, 2008; Klein and Ettinger, 2019). While often stochastic, drifts may exhibit subtle directional influences from visual or attentional factors. Neuroanatomically, they stem from tonic activity in the brainstem’s neural integrator, particularly the nucleus prepositus hypoglossi (NPH) and medial vestibular nucleus (MVN), which sustain low-frequency motor neuron firing to extraocular muscles (Cannon and Robinson, 1987; Fuchs et al., 1988). The superior colliculus (SC) modulates fixational stability, while the cerebellar flocculus and vermis fine-tune drift amplitude via feedback (Hafed et al., 2009; Arnstein et al., 2015). Drifts are involuntary and, alongside microsaccades, help maintain fixation, especially when microsaccades are limited, and contribute to retinal image motion that prevents neural adaptation, supporting continuous perception of visual detail (Engbert and Mergenthaler, 2006; Rucci and Victor, 2015).

Research investigating the respective roles of drift and microsaccades in correcting fixation disparity and stabilizing overall fixation position has developed along parallel lines. Early studies suggested that only microsaccades could adjust both binocular disparity and inaccurate fixation positions. However, later findings demonstrated that drifts also contribute to these corrections, particularly in the horizontal direction—for fixation position (Steinman et al., 1967) and fixation disparity (St.Cyr and Fender, 1969). More recent evidence indicates that both microsaccades and drifts can adjust fixation position on a timescale greater than 100 milliseconds, though only microsaccades appear to be involved in correcting fixation disparity over this relatively extended timescale (Engbert and Kliegl, 2004). The relative roles of microsaccades and drifts in maintaining stable binocular fixation were further examined by Møller et al. (2006), whose findings suggest that drift-related eye movements—known as *slow control*—primarily maintain the alignment of the visual line of sight within the foveal center during steady fixation.

A recent body of research has explored the role of inter-saccadic fixational eye movements—specifically, ocular drifts and tremors—in forming visual spatial representations (Aytekin et al., 2014; Rucci and Poletti, 2015; Poletti et al., 2015). Evidence indicates that the Brownian, or random-like, motion generated by these movements converts the static spatial information of the visual scene into a dynamic spatio-temporal signal on the retina. This movement causes retinal photoreceptors to encounter fluctuating luminance inputs, enhancing high spatial frequencies that emphasize object contours within the environment (Rucci and Victor, 2015). Thus, inter-saccadic fixational movements contribute to visual processing by encoding spatial information through temporal modulation, aiding in the extraction of features at early stages of visual processing (Rucci and Poletti, 2015; Rucci and Victor, 2015).

### 2.4 Vestibulo-ocular reflex

The vestibulo-ocular reflex (VOR) stabilizes the retinal image during head movements by producing compensatory eye movements in the direction opposite to head motion. This action maintains visual fixation on a static target in a stationary environment, thus preventing visual blurring. Laboratory research on VOR has been constrained by practical considerations, notably safety considerations that limit the range and intensity of vestibular stimuli for participants. Furthermore, laboratory protocols primarily assess passive head movements in the dark, focusing on controlled conditions in which the head is physically restrained or directly manipulated Büttner and Büttner-Ennever (2006), preventing neck proprioception and visual information to come into play. In healthy humans, passive whole-body motion using a rotating chair—with low-frequency sinusoidal oscillation or persistent rotation in one direction—or passive head rotations using a torque helmet are typically employed Collewijn and Smeets (2000); Bronstein et al. (2015).

The vestibulo-ocular reflex (VOR) is initiated when the vestibular system detects head motion, primarily through the semicircular canals and otolith organs of the inner ear. The semicircular canals sense angular acceleration resulting from rotational head movements; fluid displacement within the canals deflects hair cells in the crista ampullaris, transducing head rotation into neural signals that encode direction and velocity (Fernandez and Goldberg, 1971). In contrast, the otolith organs—the utricle and saccule—detect linear acceleration and head tilt by transducing otoconia displacement into hair cell activation, signaling translational motion and orientation relative to gravity (Angelaki and Cullen, 2008). These vestibular signals are conveyed via the vestibular nerve to the vestibular nuclei in the medulla and pons, where input from both ears and other sensory systems is integrated (Cullen, 2012). From there, signals are transmitted through the medial longitudinal fasciculus (MLF) to the oculomotor, trochlear, and abducens nuclei. This pathway drives compensatory, conjugate eye movements in the direction opposite to head motion, thereby stabilizing retinal images during movement (Leigh and Zee, 2015). The cerebellum, particularly the flocculus, nodulus, and posterior vermis, modulates the VOR by calibrating its gain and adapting reflex responses through motor learning. This allows for precise gaze stabilization even under varying head velocities, altered visual feedback, or long-term changes in sensorimotor conditions (Lisberger, 1988).

Functionally, the VOR manifests as *vestibular nystagmus*, a rhythmic pattern of compensatory slow phases interrupted by quick phases during sustained head rotations Robinson (1977); Land and Tatler (2009); Chun and Robinson (1978); Barnes (1979). The slow phase counteracts head movement by moving the eyes in the opposite direction, stabilizing the visual field. Ideally, the eye velocity during the slow phase matches the head’s velocity in the opposite direction, yielding a gain—eye velocity divided by head velocity—close to 1. The slow phase also demonstrates adaptability in response to visual or vestibular impairment, a process known as *VOR adaptation* or *gain adjustment*. For instance, when altered visual feedback is introduced, the slow phase incrementally adjusts its gain to restore stability, reflecting adaptation under changing conditions Shelhamer et al. (1992). For more details on VOR adaptation mechanisms, see the review from Schubert and Migliaccio (2019).

In contrast, the quick phase is a rapid saccadic movement that repositions the eyes centrally after the slow phase, allowing continued compensatory slow phases during sustained head rotation. Eye and head coordination during gaze orientation can follow two strategies, depending on the influence of slow and quick phases of vestibular nystagmus on eye eccentricity Lestienne et al. (1984). The first strategy, seen in highly alert animals, directs the gaze with head motion, known as the *“look where you go”* strategy. In this case, the overall eccentricity of the eye displacement in the orbit—also known as the *beating field* or *schlagfeld*—aligns with the head’s movement, as quick phases dominate the slow ones. The second strategy, *“look where you came from”*, involves directing the gaze opposite the head’s motion. Here, slow phases dominate, causing the beating field to shift contralaterally. These strategies represent the extremes of a spectrum, with intermediate patterns influenced by factors such as the level of alertness, behavioral context, and sensory-motor demands.

The VOR consists of rotational and translational components that stabilize vision during head movements. The rotational VOR compensates for angular rotations around the three principal axes, driven by semicircular canals detecting angular acceleration, ensuring near-complete visual stabilization during rapid movements Leigh and Zee (2015). The translational VOR stabilizes gaze during linear displacements—forward, backward, or lateral—via otolith organs, which detect linear acceleration and gravitational forces. However, the translational VOR is subject to limitations due to tilt-translation ambiguity, as the otolith organs respond similarly to both linear acceleration and changes in head tilt relative to gravity Angelaki and Yakusheva (2009). Resolving this ambiguity requires multimodal integration of signals from the semicircular canals, visual inputs, target distance, and image eccentricity Angelaki (1998); Paige and Tomko (1991); Telford et al. (1997). These findings suggest that the VOR is only one contributor to eye stabilization, which is based on multimodal sensory integration, which combines vestibular, visual, and proprioceptive information to optimize both precision and adaptability. Additionally, distinct VOR mechanisms are likely engaged during actively generated head movements, as opposed to passively induced ones Büttner and Büttner-Ennever (2006); Cullen and Roy (2004). These perspectives contrast with previous findings from controlled laboratory settings and will be elaborated in Section 3.1.2.

Abrupt head movements, known as *head impulses*, challenge the vestibulo-ocular reflex (VOR) to stabilize vision by producing eye movements that counteract rapid head rotations, typically at velocities of 150–300° per second. The VOR relies primarily on the inner ear’s semicircular canals (Leigh and Zee, 2015). During passive head impulses, such as when a clinician swiftly turns a patient’s head, the reflex depends almost entirely on this vestibular input, with little influence from neck muscle feedback or voluntary control. This isolation highlights the VOR’s ability to maintain gaze stability, achieving a gain—eye velocity divided by head velocity—close to 1 in healthy individuals, ensuring smooth compensatory eye movements that keep the visual world steady (Halmagyi and Curthoys, 1988). When vestibular disorders like vestibular neuritis disrupt this process, reduced gain causes the eyes to lag behind head motion, leading to retinal slip—blurred vision as the image drifts across the retina—often corrected by saccades to refocus on the target (Strupp and Brandt, 2009).

The advent of high-frequency video head impulse testing (vHIT) has transformed how clinicians evaluate VOR performance during these rapid movements. Using high-speed infrared cameras sampling at 250–500 cycles per second, vHIT captures eye and head movements with high spatial precision. This technology quantifies VOR gain and detects covert saccades—quick, involuntary eye adjustments that compensate for inadequate reflex performance—offering a sensitive measure of vestibular health (MacDougall et al., 2009) In unilateral vestibular hypofunction, such as in vestibular neuritis, vHIT reveals diminished gain and corrective saccades when the head turns toward the affected side. Bilateral vestibulopathy, often triggered by ototoxic drugs like aminoglycosides, shows severely reduced gain in both directions, resulting in oscillopsia, a disorienting visual motion that disrupts daily activities like walking (Zingler et al., 2007). Central disorders, particularly those affecting the cerebellum’s flocculus and nodulus, impair the brain’s ability to fine-tune VOR gain, leading to inconsistent eye responses across head velocities due to disrupted cerebellar modulation (Migliaccio et al., 2004; Kheradmand and Zee, 2011).

These impairments underscore the VOR’s vulnerability to disruptions in the semicircular canals, brainstem circuits, or cerebellar pathways, all of which can compromise the reflex’s ability to stabilize gaze. By pinpointing whether deficits stem from peripheral issues, like inner ear damage, or central causes, such as cerebellar lesions, vHIT provides critical diagnostic clarity, guiding tailored vestibular rehabilitation strategies to restore gaze stability (Tarnutzer et al., 2016; Sulway and Whitney, 2019).

### 2.5 Optokinetic reflex

The optokinetic reflex (OKR) is a visually mediated reflex that engages when a large segment of the visual field moves relative to the eyes, typically triggered when the surrounding environment appears to move while the observer remains stationary (Fletcher et al., 1990; Tarnutzer and Straumann, 2018; Büttner and Büttner-Ennever, 2006). This reflex primarily responds to *“retinal slip”*, the relative movement of images across the retina during both environmental and self-induced motion Fletcher et al. (1990). The OKR works synergistically with the vestibulo-ocular reflex (VOR) to process optic flow, responding either to rotational motion around the individual—rotational OKR—or to fronto-parallel translational motion—translational OKR. This synergy is especially important for low-frequency motions below 0.2 Hz, for which the gain of the VOR is low (Büttner and Büttner-Ennever, 2006; Fletcher et al., 1990; Schweigart et al., 1997; Land and Tatler, 2009). Although OKR and VOR share neural substrates, the OKR operates with a longer latency—around 150 milliseconds—due to its reliance on visual input (Land and Tatler, 2009).

The optokinetic reflex (OKR) is driven by a complex neural network involving the retina, brainstem, and cerebellum (Cohen et al., 1977; Leigh and Zee, 2015). Retinal ganglion cells detect large-field visual motion and transmit signals through the accessory optic system, including the nucleus of the optic tract (NOT) and dorsal terminal nucleus, which process directional motion cues (Simpson, 1984; Mustari and Fuchs, 1990). These brainstem structures integrate sensory input and collaborate with the vestibular nuclei to produce compensatory eye movements (BuÈttner-Ennever and Horn, 2002; Giolli et al., 2006). The cerebellum, particularly the flocculus and paraflocculus, fine-tunes OKR responses by modulating motor output based on visual feedback and predictive learning, ensuring precise gaze stabilization during head or environmental motion (Waespe et al., 1983; Voogd and Barmack, 2006).

Experimentally, the OKR is commonly induced by rotating a striped drum—known as the Bárány nystagmus drum—around the subject, who observes alternating black and white stripes or dot patterns (Fletcher et al., 1990; Distler and Hoffmann, 2011). This setup typically elicits a reflexive, oscillatory eye movement characterized by an alternating sequence of quick and slow phases (Garbutt et al., 2003; Büttner and Büttner-Ennever, 2006). Quick phases are fast, ballistic eye movements directed opposite to the direction of the visual flow. These movements share properties with ocular saccades and function to reposition the eyes toward a central orbital position, countering the visual motion stimulus (Fletcher et al., 1990; Kaminiarz et al., 2009). In contrast, the slow phases are low-velocity compensatory movements that align with the stimulus motion. The correction, however, is not perfect, as the gain—defined as the ratio of slow-phase velocity to stimulus velocity—is less than one and decreases as stimulus speed increases (Fletcher et al., 1990; Land and Tatler, 2009).

From a computational perspective, three primary models explain the alternation between quick and slow phases in optokinetic nystagmus: (i) the *eye position control hypothesis*, which suggests that quick phases are triggered to keep the eye within a certain orbital position range (Ter Braak, 1936; (ii) the *internal timing hypothesis*—or clocking model—originally proposed by Ohm (1928), which posits the existence of a central interval generator that times the onset of quick phases; and (iii) the *hybrid position-interval generator hypothesis*, which combines elements of both position control and timing regulation. Supporting evidence indicates the presence of a Gaussian-based interval generator—a biological clock—that modulates the timing of quick phases and can be influenced by concurrent cognitive tasks (Balaban and Ariel, 1992; Balaban and Furman, 2017). It’s important to note that the timing and amplitude of these phases are highly variable (Carpenter, 1993; Trillenberg et al., 2002), and the underlying cause of this variability remains not fully understood, despite extensive statistical analysis (Waddington and Harris, 2012).

Studies of the optokinetic reflex (OKR) primarily focus on its slow-phase components, often considered analogous to smooth pursuit (Robinson, 1968; Klein and Ettinger, 2019). The slow phase consists of two components: the direct component, or *ocular following response* (Büttner and Kremmyda, 2007), and the indirect component, also known as the *velocity-storage mechanism* (Raphan et al., 1979; Fletcher et al., 1990; Büttner and Büttner-Ennever, 2006). Despite similarities to smooth pursuit, these movements differ in key aspects. The direct component has a much shorter onset latency (60–70 ms) Büttner and Kremmyda (2007), is triggered by motion across a large visual field rather than a single target, and is reflexive rather than volitional. In humans, it accounts for most reflexive OKR movements at velocities up to 120°/s (Büttner and Büttner-Ennever, 2006; Büttner and Kremmyda, 2007). The indirect component, in contrast, develops gradually during sustained stimulation, integrating visual, vestibular, and somatosensory inputs to maintain slow-phase eye velocity (Raphan et al., 1979; Fletcher et al., 1990). Although the direct component dominates during initial stimulation (Van den Berg and Collewijn, 1988), the velocity-storage function of the indirect component is evident in *optokinetic after-nystagmus*, a gradually diminishing nystagmus that continues even after an abrupt transition to complete darkness, reflecting its sustained influence (Magnusson et al., 1985; Büttner and Büttner-Ennever, 2006; Tarnutzer and Straumann, 2018).

Abnormalities in the optokinetic reflex (OKR) are valuable diagnostic markers in a range of neurological and vestibular disorders. In cerebellar ataxias, particularly spinocerebellar ataxia type 1 (SCA1), OKR gain—defined as the ratio of slow-phase eye velocity to stimulus velocity—is typically reduced. The slow phases may appear irregular due to floccular dysfunction, which impairs the *velocity storage mechanism* that sustains the reflex (Leigh and Zee, 2015; Lal and Truong, 2019). These abnormalities reflect cerebellar contributions to OKR calibration and integration with vestibular signals. In vestibular neuritis, OKR responses become asymmetrical, with significantly diminished gain toward the side of the lesion. This reflects impaired visual-vestibular integration within the vestibular nuclei, particularly in the absence of peripheral vestibular input (Strupp and Brandt, 2009). Progressive supranuclear palsy (PSP), on the other hand, is associated with profound OKR impairment, especially for vertical motion stimuli, where slow-phase responses are either absent or show severely reduced gain. This is attributed to midbrain degeneration, notably involving the nucleus of the optic tract (NOT) and rostral interstitial nucleus of the medial longitudinal fasciculus (riMLF) (Chen et al., 2010; Leigh and Zee, 2015). OKR dysfunction specifically reflects compromised large-field visual motion processing and its integration with cerebellar and brainstem control systems. OKR gain and slow-phase variability, typically measured using rotating drum setups or full-field optokinetic stimulation, serve as sensitive, non-invasive biomarkers for both central and peripheral pathologies (Büttner and Kremmyda, 2007).

### 2.6 Vergence eye movements

Vergence eye movements are vital for binocular vision, enabling both eyes to align precisely on objects at varying distances. This alignment produces a single, fused visual image and supports stereoscopic depth perception, essential for activities such as reading, driving, or navigating complex environments (Leigh and Zee, 2015). Unlike saccades, which rapidly shift gaze, or smooth pursuit, which tracks moving objects, vergence involves simultaneous rotation of the eyes in opposite directions. Convergence directs the eyes inward for near objects, while divergence directs them outward for distant ones. These movements depend on four interdependent mechanisms—fusional, accommodative, proximal, and tonic—each responding to distinct visual or perceptual cues (Schor and Ciuffreda, 1983).

Fusional vergence, driven by retinal disparity, eliminates image misalignment between the eyes to maintain a single percept, proving vital for dynamic tasks like tracking moving objects (Schor and Ciuffreda, 1983). Accommodative vergence, linked to lens focusing, initiates convergence when ciliary muscles contract to sharpen a blurred image, functioning effectively even in monocular viewing or low-contrast conditions (Fincham and Walton, 1957). Proximal vergence, triggered by perceived object nearness through cues like object size or looming motion, enables rapid eye pre-alignment before disparity or blur cues fully engage, such as when approaching a book to read (Rosenfield and Rosenfield, 1997). Tonic vergence establishes a baseline eye alignment through sustained extraocular muscle tone, maintaining stable posture during rest or minimal visual stimulation (Schor, 1985). These mechanisms work together seamlessly. For instance, during reading, accommodative vergence initiates convergence to focus on text, fusional vergence fine-tunes alignment for single vision, proximal vergence adjusts to the perceived page distance, and tonic vergence ensures stable eye posture (Ciuffreda and Tannen, 1995).

A sophisticated neural network coordinates these vergence mechanisms. The midbrain supraoculomotor area, near the oculomotor nucleus, encodes vergence angle and velocity, integrating disparity, blur, and proximity cues to govern fusional, accommodative, and proximal vergence (Mays, 1984). The superior colliculus aligns vergence with saccades for smooth gaze shifts between near and far objects, while the cerebellum, through its vermis and flocculus, calibrates interactions to prevent misalignment (Gamlin, 2002). Cortical regions process complex cues: the visual cortex handles disparity for fusional vergence, the parietal cortex processes depth, and the frontal eye fields integrate vergence with saccades (Cumming and DeAngelis, 2001). The brainstem’s pontine reticular formation and pretectal area orchestrate the near triad, linking accommodative vergence to lens accommodation and pupillary constriction (Leigh and Zee, 2015). Disparity-sensitive neurons in the visual cortex drive fusional vergence, blur-sensitive pathways via the Edinger-Westphal nucleus trigger accommodative vergence, the middle temporal area processes motion and depth for proximal vergence, and sustained midbrain motor neuron activity maintains tonic vergence (Gamlin, 2002; Cumming and DeAngelis, 2001).

Physiologically, vergence relies on extraocular muscles. The medial rectus muscles, controlled by the oculomotor nerve, power convergence, while the lateral rectus muscles, controlled by the abducens nerve, facilitate divergence (Von Noorden, 1996). Vergence operates more slowly than saccades, achieving velocities of 10–20° per second with latencies of 160–200 milliseconds, relying on visual feedback from disparity and blur (Leigh and Zee, 2015). The near triad integrates accommodative vergence with autonomic processes: accommodation sharpens focus through ciliary muscle contraction, and pupillary constriction enhances depth of field via the sphincter pupillae (Von Noorden, 1996). Fusional vergence employs fine motor adjustments to align retinal images, proximal vergence initiates broader movements based on perceptual cues, and tonic vergence maintains stability through muscle spindle feedback (Schor, 1985). In young, healthy adults, convergence amplitude typically reaches 25 to 30 prism diopters—a unit measuring eye deviation—while divergence amplitude ranges from 6 to 10 prism diopters, reflecting the greater physiological demand for near vision. These amplitudes may decrease with age or in pathological conditions (Hung et al., 1986).

Clinical evaluation of vergence is crucial for diagnosing binocular vision disorders. The near point of convergence test measures the closest point at which the eyes maintain single binocular vision, typically 5–10 cm, assessing fusional and accommodative vergence. A receded near point often indicates convergence insufficiency (Scheiman et al., 2003). Vergence facility testing evaluates the ability to alternate efficiently between convergence and divergence, reflecting the adaptability of fusional and proximal vergence during prolonged near tasks (Gall et al., 1998). Prism vergence testing, using base-in and base-out prisms, quantifies the range of fusional vergence, determining the maximum disparity overcome before double vision occurs, critical for assessing compensatory capacity in latent deviations—phorias (Evans, 2021). The cover test, performed unilaterally or alternately, detects phorias and tropias by evaluating alignment under dissociated viewing conditions, revealing deficits in tonic vergence or overall coordination (Von Noorden, 1996).

Common dysfunctions include convergence insufficiency, marked by eye strain, intermittent double vision, headaches, or difficulty sustaining attention during reading or screen use, often due to impaired fusional or accommodative vergence (Scheiman et al., 2003). Other abnormalities, such as excessive convergence, limited divergence, or vergence paralysis, may stem from midbrain lesions, strabismus, or uncorrected refractive errors (Leigh and Zee, 2015). Management strategies include vision therapy targeting the deficient mechanism, prism lenses to aid compensation, or orthoptic exercises to enhance fusional reserves and vergence facility. These approaches, particularly effective for convergence insufficiency, are supported by clinical evidence (Scheiman et al., 2005; Scheiman and Wick, 2008).

## 3 Ecological conditions

First and foremost, the term ecological must be nuanced. Rather than striving for ecological validity in its broadest sense—an evolving concept across cognitive sciences and neurophysiology (Holleman et al., 2020) — the focus here is on experimental paradigms that do not impose physical constraints on the observer’s body. Under such conditions, gaze reorientation involves coordinated movements of not only the eyes but also the head, trunk, and feet (Anastasopoulos et al., 2009; Land, 2004). In contrast to tightly controlled environments—particularly experimental settings that involve physical head restraint, where gaze is typically studied in isolation—natural gaze behavior arises from a dynamic system that integrates vestibular, proprioceptive, and visual inputs into task-specific motor outputs.

To investigate eye movements in unconstrained settings, most studies have focused on eye-head coordination, a specific subset of the broader problem (Zangermeister and Stark, 1982; Afanador et al., 1986; Fuller, 1992; Guitton, 1992; Stahl, 1999; Stahl, 2001; Pelz et al., 2001; Einhäuser et al., 2007; Thumser et al., 2008). Coordinating gaze shifts with head movements introduces additional complexity, even within this more constrained framework (Freedman, 2008). posed key questions regarding eye-head coordination: *”Are the eye and head components of gaze shifts tightly linked, or are they dissociable? What factors determine the extent of head involvement? […] When the head contributes to gaze shifts, it moves concurrently and in the same direction as the eyes, so what role does the vestibulo-ocular reflex (VOR) play?”* While these issues have been extensively explored and numerous hypotheses have been proposed, they remain subjects of active investigation.

### 3.1 Gaze stabilizing movements

Although maintaining a relatively stable retinal image is crucial for high visual acuity, the human head is almost always in motion. Consequently, tasks that demand fine visual focus, such as reading, would be unfeasible without robust compensatory systems to offset these head movements. Fortunately, a number of mechanisms come into play to provide a stable gaze.

#### 3.1.1 Fixational eye movements

The role—and existence as canonical constituent of eye movements—of microsaccades is even more contentious under natural viewing conditions than when the head is restrained. Some researchers posit that microsaccades contribute to visual attention (Fischer and Weber, 1993b), enhance visual processing (Melloni et al., 2009), or may indicate levels of concentration (Buettner et al., 2019). However, others have observed that microsaccades are exceedingly rare in real-world activities. Malinov et al. (2000), for instance, analyzed eye movements during a naturalistic task and found that only 2 of the 3,375 saccades recorded could be classified as microsaccades. As Collewijn and Kowler (2008) summarize: *“A special role for microsaccades seemed particularly unlikely to emerge under natural conditions, when head movements are permitted during either fixation or during the performance of active visual tasks.”*

On the other hand, the precise measurement of fine eye movements, including ocular drift and micro-tremor, under natural conditions only became feasible in the 1990s with the development (Edwards et al., 1994) of the Maryland Revolving-Field Monitor (MRFM). To our knowledge, the MRFM remains the only eye tracker with a precision demonstrated to be sufficient to record these fixational eye movements during normal head movements (Aytekin et al., 2014; Rucci and Poletti, 2015). This field of research thus constitutes somewhat of a niche reserved for a few laboratories with such a set-up. However, a limitation of the MRFM system is the requirement that participants remain within the magnetic field of the device, restricting studies to tasks that involve minimal body movement.

Furthermore, during unconstrained fixation, ocular drift appears anticorrelated with involuntary head movements (Aytekin et al., 2014), effectively compensating—and even anticipating (Poletti et al., 2015) — for the fixational instability of the head (Aytekin et al., 2014). This compensation, however, is only partial (Poletti et al., 2015), allowing to maintain retinal image motion close to those experienced when the head is restrained (Poletti et al., 2015). As a result, the retinal stimulation produced by fixational head and eye movements in natural conditions retains key characteristics of the signal observed in head-fixed ocular drift, including correlated temporal structures and similar spatio-temporal retinal stimulation patterns (Roberts et al., 2013; Rucci and Poletti, 2015).

#### 3.1.2 VOR and OKR

Introduced in Sections 2.4 and 2.5, respectively, the Vestibulo-Ocular Reflex (VOR) and Optokinetic Reflex (OKR) are two fundamental eye stabilizing mechanisms that act to maintain retinal image stability during body movements. In brief, the VOR counteracts head movements within a stationary environment, while the OKR compensates for movements within the visual field (Robinson, 1968). In practice, both mechanisms operate in tandem to achieve visual stability. Indeed, head movements are inevitably present during natural viewing—the VOR is thus highly active—generating vestibular inputs but also displacement of the visual field (Fletcher et al., 1990). Therefore, although not predominant compared to VOR (Pelisson et al., 1988) and with a higher latency (Collewijn and Smeets, 2000), the OKR is at least partly active as well. In sum, VOR and OKR interact naturally through visuovestibular mechanisms (Green, 2003), a phenomenon known as *visually enhanced VOR*, which has garnered substantial clinical interest for its potential applications (Arriaga et al., 2006; Szmulewicz et al., 2014; Rey-Martinez et al., 2018; Halmágyi et al., 2022).

Investigations of VOR and OKR in contexts of free head and body motion have revealed their multi-modal nature, *i.e.*, numerous indirect sensory modalities are implicated in the neural circuits underlying these reflexes, suggesting a need for holistic analysis. For instance, primate studies found that neck muscle proprioception, activated during head movement, projects to neurons within the vestibular nuclei (Gdowski and McCrea, 2000). Furthermore, there is evidence that active head movement may lead to partial suppression of vestibular input through extra-retinal mechanisms (Roy and Cullen, 2004). For instance, abrupt head movements—known as head impulses—reveal differences between active—self-generated—and passive—externally applied—responses. In passive head impulses, such as those delivered by a clinician rotating the head, the VOR relies heavily on vestibular input, with minimal contribution from neck proprioception or voluntary control, making it a sensitive measure of vestibular function (Halmagyi and Curthoys, 1988). Active head impulses, where individuals initiate their own rapid head turns, engage additional mechanisms, including pre-programmed motor commands and cervical proprioceptive feedback, which can enhance VOR gain and reduce latency compared to passive conditions (Cullen and Roy, 2004).

Beyond vestibular input, active impulses incorporate pre-programmed motor commands from the brain’s motor cortex and feedback from cervical proprioceptors, which sense neck muscle activity. These contributions can increase VOR gain and reduce response latency compared to passive conditions, reflecting the brain’s ability to predict and optimize eye-head coordination (Cullen and Roy, 2004). For example, during active impulses, healthy individuals may achieve gains slightly above 1, as predictive mechanisms anticipate head motion, ensuring seamless gaze stabilization.

Interestingly, during locomotion, studies report distinct compensatory roles for rotational and translational VOR components. Specifically, rotational head movements are fully compensated by the VOR, while translational motion is stabilized only within a fixation plane, such that objects in front of this plane exhibit relative motion opposite to the translation direction (Miles, 1998; Miles, 1997). This limitation implies that simultaneous stabilization of near and far objects is not achievable. Subsequent research suggests that, during ambulation, the brain resolves this through an optimized stabilization plane that maximizes visual clarity over distances (Zee et al., 2017).

Furthermore, the incorporation of extra-vestibular information into early vestibular processing enables VOR modulation based on behavioral goals. For instance, the VOR remains robust across a range of velocities and frequencies when gaze stabilization is the primary objective. However, during intentional gaze shifts, an efference copy of the motor command temporarily suppresses the VOR (Laurutis and Robinson, 1986; Cullen, 2019). Nonetheless, vestibular feedback remains accessible to the oculomotor system, as demonstrated by the rapid recovery of VOR function following mechanical perturbations of the head during gaze shifts (Freedman, 2008; Boulanger et al., 2012). This dynamic inhibition of the VOR is thought to be a function of the gaze error, defined as the disparity between intended and actual gaze positions (Pelisson et al., 1988; Boulanger et al., 2012).

An intriguing area in the study of nystagmus fast phases is the phenomenon of the *beating field* shift. Specifically, research has shown (Watanabe, 2001) that during optokinetic nystagmus, the average gaze position, or *beating field*, shifts in the direction of the fast phases—meaning it moves opposite to the motion of the visual field. This shift has been observed not only in humans (Abadi et al., 1999; Watanabe, 2001) but also across multiple species (Schweigart, 1995; Bähring et al., 1994). A similar directional shift occurs during vestibular nystagmus, where the mean eye position shifts in the direction of head rotation (Vidal et al., 1982; Chun and Robinson, 1978).

Observations in optokinetic and vestibular nystagmus suggest that the *beating field shift* may be a goal-directed involuntary response, acting as a reflexive orienting mechanism toward a *center of interest* (Crommelinck et al., 1982; Vidal et al., 1982; Siegler et al., 1998). This shift likely helps align gaze with self-motion, enhancing target detection within the moving visual field. Siegler et al. (1998) proposed that cognitive factors influence its magnitude, reflecting an individual’s preference for allocentric or egocentric reference frames. Additionally, proprioceptive feedback modulates the beating field by adjusting fast phase amplitude and frequency during nystagmus (Botti et al., 2001).

### 3.2 Gaze orienting movements

In this section, we overview the mechanisms that enable foveal reorientation in ecological conditions. As we will discuss, the involvement of head and sometimes hand movements adds complexity to understanding these processes.

#### 3.2.1 Gaze shifts

Under natural conditions, while gaze-orienting eye movements can occur without significant head or body segment involvement (Freedman, 2008), head movements frequently accompany gaze shifts (Pelz et al., 2001), even for small gaze amplitudes, such as those observed during reading tasks (Kowler et al., 1992; Lee, 1999). Importantly, for large-amplitude gaze shifts, coordinated movements between the eyes and head are necessary. Fuller (1992) observed that head movements were essential for horizontal gaze shifts exceeding 40°. Below this threshold, individual differences emerged, with some participants showing a tendency to move their heads with each gaze shift, reflecting an intrinsic behavioral inclination towards head involvement in gaze changes. This variability led to the categorization of individuals as *head movers* and *non-head movers* (Fuller, 1992; Afanador et al., 1986; Stahl, 1999).

Interestingly, individual predisposition for head movement during gaze shifts was not associated with differences in ocular motor control impairments at high eccentricities (Stahl, 2001). Instead, this tendency to activate the head during gaze shifts appears to be linked to the innate representation of visual space in the central nervous system (Fuller, 1992). Several factors influence the extent of head movement in gaze shifts, including the initial eccentricity of the eyes within their orbits. When the eyes are offset in the same direction as the intended gaze shift, head contribution tends to increase, and the opposite occurs when the offset is in the opposite direction (Freedman, 2008). Furthermore, head dynamics also impact eye movement properties; for instance, ocular saccade amplitude is inversely related to head velocity, with faster head movements resulting in smaller saccades (Guitton and Volle, 1987).

It might be hypothesized that the intrinsic properties of saccadic eye movements, such as the *main sequence*—the relationship between saccade amplitude, duration, and peak velocity—remain unchanged during combined eye-head gaze shifts. However, evidence reveals significant interactions between saccades and concurrent head movements that modify saccade kinematics, particularly the peak velocity-amplitude relationship. In head-free conditions, the peak velocity of saccades is often reduced compared to head-fixed saccades of the same amplitude, as the vestibulo-ocular reflex (VOR), which stabilizes gaze during head movement, interacts with the saccadic system to coordinate eye and head motion (Freedman and Sparks, 1997). While the main sequence relationship generally holds, the slope or scaling of the velocity-amplitude curve is altered, reflecting modified saccade dynamics influenced by head movement. Additionally, studies show that for horizontal gaze shifts with eyes and head aligned, saccade amplitude increases linearly for small gaze shifts but plateaus as head contribution grows for larger shifts (Guitton and Volle, 1987; Stahl, 1999). While this amplitude saturation could theoretically result from mechanical constraints of the eyes within the orbits, experimental data indicate that recorded saccade amplitudes rarely approach the physical limits of the orbital range (Guitton and Volle, 1987; Phillips et al., 1995; Freedman and Sparks, 1997).

From a descriptive perspective, eye-head coordination typically begins with a rapid saccadic eye movement toward the object of interest, immediately followed by a head movement in the same direction (Bartz, 1966; Barnes, 1979; Pelisson et al., 1988; Boulanger et al., 2012). This coordination results in a characteristic sequence as outlined by Freedman (2008). The gaze shift initiates with a high-velocity saccade—approximately 200−−400 degrees per second (Barnes, 1979) — of large amplitude, repositioning the eyes relative to the head. This fast phase generally concludes as the line of sight aligns with the target, at which point the eyes are offset in the orbits by roughly 30° while the head has moved less than 2°. Following this initial saccadic phase, the head continues to rotate for an additional 250 milliseconds, covering about 15 more degrees. During this ongoing head movement, the vestibulo-ocular reflex (VOR) compensates by counter-rotating the eyes in the opposite direction, thereby maintaining the gaze on the target. This VOR action minimizes changes in the line of sight, stabilizing the gaze despite the continued head motion.

This sequence typically introduces a delay of 25–75 milliseconds between eye and head movement onset (Zangermeister and Stark, 1981; Zangermeister and Stark, 1982; Freedman, 2008). This delay is thought to result from the greater visco-inertial load on the neck muscles compared to the lower visco-elastic resistance required for eye movement (Zangermeister and Stark, 1981; Zangermeister and Stark, 1982). Electromyography (EMG) studies show that neck muscles exhibit an increase in agonist activity and a decrease in antagonist activity about 20 milliseconds before a similar change in eye muscle EMG activity (Bizzi et al., 1972; Zangemeister and Stark, 1981). These findings suggest that neural signals for coordinated eye-head movements are first dispatched to neck muscles, followed shortly by eye muscles (Bizzi et al., 1972).

This raises the question: *Could synchronous eye-head movements be driven by a common, shared motor command?* This idea has intrigued researchers. Lestienne et al. (1984) highlighted the close coupling between saccadic eye and attempted head movements, shown by neck muscle EMG in head-restrained subjects. They suggested that while eye-head coupling may not be mandatory in primates, it likely serves as a mechanism for coordination, particularly involving reticulo-spinal neurons (Vidal et al., 1983). Further studies have shown that the covariance of eye and head movement velocities, the timing correlation of latencies, and the linear phase-plane relationship between head acceleration and eye velocity during rapid gaze shifts support the hypothesis of a shared motor command driving both movements (Guitton et al., 1990; Galiana and Guitton, 1992)

Despite the strong coupling between eye and head movements and the possibility of a shared motor command, numerous studies show that eye and head movements can be initiated separately. The timing of these movements is influenced by factors such as target predictability (Bizzi et al., 1972; Zangemeister and Stark, 1982), gaze shift amplitude (Barnes, 1979; Guitton and Volle, 1987; Freedman and Sparks, 1997), and individual tendencies for head movement (Stahl, 1999). For instance, in non-human primates, as gaze shift amplitude increases, the time from saccade onset to head movement onset decreases, eventually reaching synchrony or even showing head movement preceding saccades (Freedman and Sparks, 1997). Similar findings in humans show that head movements can sometimes precede saccades, particularly when the target is predictable (Moschner and Zangemeister, 1993). Moreover, experimentally delaying saccadic onset by stimulating the pontine omnipause neurons does not affect head movement initiation, further supporting the partial independence of eye and head command signals in brainstem structures governing coordinated eye-head actions.

Yet, despite the relative independence of eye and head command signals, gaze itself—the sum of eye and head contributions—remains tightly controlled, preserving accuracy throughout movement. This precision holds even when the head is subjected to perturbations during its trajectory (Guitton and Volle, 1987; Boulanger et al., 2012). These observations have led some researchers to propose a gaze-feedback model in which VOR-saccade interactions are guided by a gaze-error signal (Guitton and Volle, 1987; Boulanger et al., 2012).

Another important factor modulating eye-head coordination is the initial eccentricity of the eyes relative to the head at the start of a gaze shift (Abel et al., 1979; Laurutis and Robinson, 1986; Freedman, 2008). The initial eye eccentricity not only affects the head’s contribution to the gaze shift but also alters the characteristics of the eye movements. Specifically, when the eyes are offset in the direction of the gaze shift, saccadic velocity decreases compared to when the eyes are centered or offset in the opposite direction (Laurutis and Robinson, 1986). In other words, centrifugal saccades are slower than centripetal ones. Additionally, as the head’s role in the gaze shift increases, the saccadic velocity component decreases (Freedman, 2008)

Similar to eye-head coordination, eye-hand coordination is crucial for interacting with the environment. When humans reach to grasp objects, they typically initiate saccadic eye movements toward the target, followed by guiding the hand to the center of gaze at the moment of grasp (Hayhoe et al., 2002; Johansson et al., 2001). The eyes usually fixate on the object just before or shortly after the initiation of hand movement, well before the hand reaches the target (Helsen et al., 2000; Starkes et al., 2002), and maintain fixation until the movement is completed, even during sequential tasks (Neggers and Bekkering, 2000; Neggers and Bekkering, 2002). However, as in eye-head coordination, there is considerable variability in the timing of eye and hand movements during natural tasks (Abrams et al., 1990; Carnahan and Marteniuk, 1991; Pelz et al., 2001).

In the broader context of multi-segment coordination, eye-head movement patterns adapt when additional body segments are involved. During reaching and pointing tasks, Carnahan and Marteniuk (1991) found that the timing of eye and head movements varied based on task demands. For rapid pointing, head movement began before the eyes, whereas in precision tasks, the eyes moved first. In trials without hand movements, the eyes consistently initiated movement before the head, suggesting that the involvement of additional body segments and higher-level cognitive factors can modulate eye-head coordination.

#### 3.2.2 Gaze pursuits

Most studies on ocular pursuit have been conducted under conditions where the head is restrained, allowing only eye movements to track a moving target. However, in real-world scenarios, humans track moving targets through a combination of eye, head, and body movements (Lanman et al., 1978; Ackerley and Barnes, 2011b; Pallus and Freedman, 2016). To date, no studies have fully investigated the mechanisms of visual pursuits under conditions of complete freedom of body movements. Instead, research has typically focused on the more restricted context of eye-head coordination during head-free gaze pursuits, both in humans and non-human primates. Despite this narrower focus, publications on this topic remain limited. As early as 1989, Barnes noted the scarcity of studies on head-free pursuit, citing only six articles from the previous 2 decades (Barnes and Lawson, 1989). Over 30 years later, this observation still holds true, with only a small number of additional studies contributing to the literature (Collins and Barnes, 1999; Dubrovsky and Cullen, 2002; Barnes and Collins, 2008b; Barnes and Collins, 2008a; Ackerley and Barnes, 2011b; Ackerley and Barnes, 2011a; Pallus and Freedman, 2016; Shanidze and Velisar, 2020).

Head-free pursuits differ from head-restrained ones by requiring the integration of visual, vestibular, and neck proprioceptive signals to control both the eyes and the head (Lanman et al., 1978; Dubrovsky and Cullen, 2002). Gaze pursuits involve both retinal and extra-retinal inputs, with their contributions evolving over time. Like head-restrained conditions, head-free gaze pursuits consist of an initiation phase, driven by retinal slip signals during the first 80−100 milliseconds, followed by a maintenance phase. In this phase, extra-retinal inputs, including neck proprioception, vestibular signals, attention, expectation, and efferent copies of eye and head movements, are integrated with visual signals to sustain pursuit. Pursuit velocity aligns with the target’s velocity, typically reaching equilibrium within 200–300 milliseconds (Barnes and Collins, 2008a)

The role of extra-retinal inputs in maintaining gaze pursuit was demonstrated in extinction paradigms, where removal of the pursuit target shortly after the initiation phase did not immediately disrupt the pursuit. Depending on factors such as the duration of initial target exposure and expectations regarding the target’s reappearance, participants could continue pursuing the target with appropriate direction and velocity (Ackerley and Barnes, 2011b; Ackerley and Barnes, 2011a). These findings suggest that the pursuit system forms an internal representation of the target’s motion and velocity, which it uses to continue pursuit even without direct visual feedback. This *memory* of target velocity (Barnes and Collins, 2008b) is likely communicated to the eye and neck motor systems—potentially with different controller parameters to account for the distinct biomechanical properties of the eyes and head—and is thus a key component of eye-head coordination during gaze pursuits (Dubrovsky and Cullen, 2002; Pallus and Freedman, 2016).

This close coordination between the eyes and head ensures that gaze characteristics, such as position and velocity, are nearly identical under both head-restrained and head-unrestrained conditions (Ackerley and Barnes, 2011b; Pallus and Freedman, 2016). Pursuit gain—defined as the ratio of gaze displacement to target displacement—remains near unity in both conditions, with the exception of older individuals, who exhibit a slight decrease in pursuit gain when the head is unrestrained (Shanidze and Velisar, 2020). Despite the lack of measurable advantage in terms of accuracy, head movements are consistently coupled with eye movements during pursuit of targets with both predictable and unpredictable trajectories (Lanman et al., 1978; Ackerley and Barnes, 2011b). This is thought to help keep the eyes centered within their orbits, ensuring that any subsequent eye movements toward a secondary point of interest can make use of the full oculomotor range (Dubrovsky and Cullen, 2002; Pallus and Freedman, 2016). For instance, Dubrovsky and Cullen (2002) found that the eyes generally remained within 15 degrees of eccentricity during pursuit in non-human primates.

However, this reductionist explanation does not fully capture the considerable variability in strategies used during gaze pursuit (Dubrovsky and Cullen, 2002; Pallus and Freedman, 2016). In some cases, eye and head movements can be entirely uncoupled (Pallus and Freedman, 2016; Collins and Barnes, 1999). In most situations, however, an ocular saccade is first executed toward the target to initiate the pursuit, followed by the recruitment of head movements after a brief delay (Ackerley and Barnes, 2011b; Shanidze and Velisar, 2020). This is typically followed by a coordinated but variable combination of eye and head movements to maintain pursuit (Pallus and Freedman, 2016; Shanidze and Velisar, 2020), with the head often accounting for the majority of the gaze displacement, albeit with significant inter-subject variability (Lanman et al., 1978; Ackerley and Barnes, 2011a).

During pursuit maintenance, head trajectory or velocity may deliberately diverge from target motion (Collins and Barnes, 1999; Pallus and Freedman, 2016). Variations in head movement are almost immediately compensated by eye movements, minimizing gaze tracking error (Collins and Barnes, 1999). This compensation is thought to arise from an internal gaze-related signal in the central nervous system, incorporating stored information about target velocity (Collins and Barnes, 1999; Dubrovsky and Cullen, 2002). While some researchers describe gaze pursuit as a sequence of discrete saccades and smooth pursuits (Pallus and Freedman, 2016; Shanidze and Velisar, 2020), others have identified compensatory mechanisms for head motion within the eye movement trace (Ackerley and Barnes, 2011a; Shanidze and Velisar, 2020). These findings suggest that the VOR remains active during gaze pursuit, modulated by visual feedback and extra-retinal signals (Ackerley and Barnes, 2011a)

## 4 Practical considerations

While the physiological insights discussed thus far highlight the complexity of gaze control, understanding the different methodologies used to record eye and head movements is essential for avoiding potential pitfalls when selecting recording equipment and experimental paradigms.

### 4.1 Eye movement measurement

In this section, we provide a brief overview of eye movement measurement techniques, highlighting their key characteristics. For more detailed reviews on the history, methods, and techniques of eye tracking, we refer to more comprehensive sources (Young and Sheena, 1975; Wade, 2007; Hansen and Ji, 2009; Holmqvist et al., 2011; Yarbus, 2013; Chennamma and Yuan, 2013; Cognolato et al., 2018).

#### 4.1.1 Electro-Oculography (EOG)

Electro-Oculography (EOG) measures electric potential differences using electrodes positioned near the orbital margins, with pairs placed close to the medial and lateral canthi for horizontal movements and above and below the eyes for vertical movements, to track eye movements. By detecting changes in the corneo-retinal standing potential, EOG enables independent monitoring of horizontal and vertical eye movements, making it valuable for clinical applications such as sleep studies where eye closure occurs (Barea et al., 2002). Its advantages include affordability, ease of implementation, and the ability to function with closed eyes, which is critical for diagnosing sleep disorders. However, EOG has significant limitations. It is primarily suited for controlled laboratory conditions due to the need for stable electrode contact, which is disrupted by motion, sweat, or skin movement in dynamic environments (Bulling et al., 2009). Signal noise from facial muscle activity or external electrical interference reduces accuracy, and frequent recalibration is required due to signal drift (Barea et al., 2002). While wearable EOG devices have been explored for monitoring eye movements during daily activities (Bulling et al., 2009), their practicality is limited by lower spatial resolution compared to other methods and challenges in maintaining reliable electrode placement (Holmqvist and Andersson, 2017). As a result, EOG is less suitable for field applications and has been largely replaced by non-invasive techniques.

#### 4.1.2 Scleral contact lens/search coil systems

Scleral contact lens/search coil systems use a wire coil embedded in a contact lens worn on the sclera, which moves within a controlled magnetic field to induce an electric current proportional to eye position. Evolving from early mechanical methods that fixed markers directly on the cornea (Delabarre, 1898), modern scleral coils provide exceptional accuracy and high temporal resolution, historically serving as a reference standard for calibrating other eye-tracking systems (Young and Sheena, 1975). However, the method’s invasive nature causes significant discomfort and poses risks such as corneal irritation or infection, restricting session durations to typically under 30 min (Holmqvist and Andersson, 2017). Operation within a Faraday cage is necessary to shield against electromagnetic interference, confining use to specialized laboratory settings and prohibiting mobility (Young and Sheena, 1975). The complex setup, involving magnetic fields and precise calibration, is costly and inaccessible for most applications. Due to these limitations, scleral coil systems are increasingly obsolete, replaced by non-invasive methods like video-oculography for most research and practical purposes (Holmqvist and Andersson, 2017). Their use persists only in niche calibration tasks.

#### 4.1.3 Infrared Oculography (IROG)

Infrared Oculography (IROG) tracks eye movements by measuring the intensity of reflected infrared light, often using dual Purkinje imaging (DPI). DPI compares reflections from the cornea’s anterior surface—first Purkinje image—and the lens’s posterior surface—fourth Purkinje image—which shift relative to each other as the eye rotates, providing measurements robust to eye translation (Cornsweet and Crane, 1973). IROG offers high spatial and temporal resolution, making it suitable for precise laboratory studies (Crane and Steele, 1985). However, its limitations are notable. Precise head stabilization, typically via chinrests or head mounts, is required to maintain alignment with infrared cameras, restricting applications to controlled environments (Cornsweet and Crane, 1973). The method is limited to a visual angle of approximately 15° from the center, as the fourth Purkinje image becomes occluded by the iris at larger angles, reducing accuracy (Crane and Steele, 1985). Sensitivity to ambient infrared light or reflective surfaces, such as glasses, can degrade performance, necessitating controlled lighting conditions (Holmqvist and Andersson, 2017). The complex setup and calibration requirements further increase cost and user effort, making IROG less practical for mobile or real-world applications compared to video-based systems.

#### 4.1.4 Video-based eye tracking (VOG)

Video-based Eye Tracking, or Video-Oculography (VOG), uses one or more cameras, typically with near-infrared light, to capture eye appearance and track features like the pupil and corneal reflection—first Purkinje image. By computing the vector between the pupil center and corneal reflection, VOG maps eye positions to points in the visual field (Hansen and Ji, 2009). Its non-invasive nature and flexibility make it the most widely used eye-tracking method, supporting variants such as limbus or iris-sclera boundary tracking (Cognolato et al., 2018). A significant limitation is its reliance on a calibration procedure, where participants fixate on predefined targets to establish a mapping between eye features and gaze points, which is time-consuming and requires cooperation (Hansen and Ji, 2009). VOG is sensitive to environmental factors, including ambient light, occlusions (e.g., eyelids, glasses), and reflections, which can reduce accuracy in uncontrolled settings (Holmqvist and Andersson, 2017). Significant head movements disrupt tracking, necessitating stabilization or additional compensation techniques Cognolato et al. (2018). Accuracy also decreases at large visual angles—*e.g.*, beyond 30°—due to pupil distortion or occlusion (Hansen and Ji, 2009). Despite these challenges, VOG remains the most versatile and practical eye-tracking method for both research and commercial applications.

EOG, scleral contact lens/research coil, and IROG are specialized methods used in controlled environments for high-precision research, particularly in neurophysiology, vision, and ophthalmology. In contrast, VOG is easier to implement and has become the dominant eye-tracking technique, widely used in commercial systems. While eye-gaze tracking was once complex and expensive, recent advancements have lowered costs and improved VOG efficiency. Unlike other systems requiring specialized training, VOG is accessible to a broader range of researchers. However, with many commercial options available, selecting the right equipment can be challenging. To assist in this, we review the technical specifications of VOG systems and discuss their applicability to different experimental paradigms in the subsequent sections.

### 4.2 Technical specifications

A typical VOG eye tracker comprises a camera, a lighting system, and software for detecting and tracking eye movements. A key metric for evaluating eye-tracker spatial quality is *gaze accuracy*, defined as the average angular distance between the true and recorded gaze positions, with smaller distances indicating better accuracy (Feit et al., 2017). *Spatial resolution*, related to accuracy, refers to the smallest detectable eye movement, while *gaze precision* measures the consistency of gaze position over time, often quantified as the root mean square sample-to-sample (RMS-s2s) deviation. Accuracy and precision are typically assessed separately in horizontal and vertical directions, though manufacturers often provide a single aggregated value for each.

System *latency* refers to the delay between actual eye movement and the corresponding time reported by the eye tracker, while variability in latency is described as temporal precision (Holmqvist et al., 2012). This latency can be critical in certain experimental setups, particularly in gaze-contingent tasks where visual stimuli dynamically update based on gaze position. End-to-end latency encompasses multiple factors, including camera exposure time, image readout and transfer, processing delays, data transmission, and display refresh rate.

*Data loss*, on the other hand, indicates the proportion of samples during which no gaze coordinate was reported compared to the reported sampling rate of the eye tracker. These losses could result from blinks or from the recording device’s inability to effectively track eye movements, particularly for eccentric eye movements. Also, it is sometimes desirable to differentiate blinks from other sources of data loss. For instance, this differentiation may be necessary when blinks serve as a behavioral measure (Leal and Vrij, 2008) or as inputs for gaze-based interactions, such as selection inputs.

Lastly, the *sampling frequency* of an eye tracking system denotes how often the eye tracker records the position of the eyes per second. A higher sampling frequency enhances the accuracy of estimating the actual trajectory of eye movements. However, this increased frequency comes with certain drawbacks, such as the need for more expensive cameras, higher illumination requirements, possibly increased levels of noise, and ultimately, a larger amount of data to be stored.

Standardized criteria for reporting eye-tracking data quality are lacking, with many studies relying on manufacturer specifications. However, discrepancies often exist between reported accuracy and actual performance, even in controlled conditions (Nyström et al., 2013a; Blignaut et al., 2014). This highlights the need for standardized quality assessments and independent validation, similar to practices in other technology fields. Benchmarking eye-tracking systems against a gold standard, such as the Scleral Contact Lens or Search Coil System, is essential for ensuring reliability. For detailed guidance on data quality assessment, see Holmqvist et al. (2012).

### 4.3 Eye-trackers and experimental paradigms

This section focuses on video-based eye trackers, as other methods in Section 4.1 serve specialized needs. Video-based systems fall into four types: fixed, remote, wearable, and integrated. The following sections outline each, with their strengths and limitations. Table 1 summarizes the main types of video-based eye trackers, highlighting their typical applications and limitations. Table 2 provides an overview of commercially available devices with key technical specifications.

**TABLE 1:** Summary of main types of video-based eye-tracking devices, together with typical usages and main limitations.

Type of device	Example of use	Main limitations
Fixed/Head Stabilized	• Screen-based visual tasks • High-precision research in neuroscience and phhysiology exploring oculometric dynamics	• Commonly requires head stabilization • Limited to laboratory conditions • Unsuitable for certain populations
Remote	• Screen-based visual tasks • Research in neuroscience, cognitive science, psychology and marketing • Participants with neurological impairments, infants	• Viewer must stay within the device headbox • Excessive head and body movements can cause inaccuracy and data loss • Generally not suitable for analyzing fine oculomotor dynamics
Wearable	• Behavioral experiments outside the laboratory • Research in neuroscience, human factors, ergonomics and marketing • Driving experiments and high-fidelity simulators	• Data recorded relatively to the viewer field of view, requiring further analysis • Interpreting the results is more complicated due to increased viewer freedom • Generally not suitable for analyzing fine oculomotor dynamics
Integrated	• Augmented/virtual reality research in neuroscience, cognitive science and psychology • Medical analysis tools	• Generally highly-specialized to a specific use-case • Participant has to tolerate augmented/virtual reality

**TABLE 2:** This comparison focuses on the technical specifications of commercial video-based eye-trackers. When available, the specifications are those provided by the manufacturers; otherwise, the data presented are based on observations reported in the research community. Notably, we include the *SMI ETG 2*, a video-based eye tracker frequently referenced in the literature. Although this device has been widely used in various studies, it has been discontinued following Apple’s acquisition of SMI in 2017.

Device	Type	Accuracy (*deg*)	Sampling rate (*Hz*)	Head-box (*cm*)	Latency (*ms*)	Price point ($)
Arrington ViewPoint	Tower-mounted	0.3−1 (accuracy) 0.25 (precision)	90−400	—	<20	<15000
Tobii Pro Spectrum	Tower-mounted	0.3−0.4 (accuracy) 0.06 (precision)	60−1200	34×26 at 65cm (free-head)	<10	>15000
EyeLink 1000 Plus	Tower-mounted	0.3−0.5 (accuracy) 0.01 (fixed-head precision) 0.05 (free-head precision)	250−2000 (fixed-head) 250−1000 (free-head)	40×40 at 70cm (free-head)	<10	>15000
Gazepoint GP3	Remote	0.5−1 (accuracy) 0.1 (precision)	60−150	35×22	<50	<5000
Tobii Pro Spark	Remote	0.5 (accuracy) 0.3 (precision)	33−60	35×35 at 65cm	<50	<5000
EyeLogic LogicOne	Remote	0.5 (accuracy) 0.1 (precision)	60−250	30×20 at 60cm	<20	<15000
SmartEye Aurora	Remote	0.3 (accuracy) 0.1 (precision)	60−250	50×40 at 65cm	<20	<15000
Tobii ProGlasses 2	Wearable	1.4 (accuracy) MacInnes et al. (2018b) 0.3 (precision) MacInnes et al. (2018b)	50−100	—	<50	<15000
SMI ETG 2	Wearable	0.5−1 (accuracy) MacInnes et al. (2018b) 0.2 (precision) MacInnes et al. (2018b)	60−120	—	<50	<15000
PupilLabs Core	Wearable	0.6 (accuracy) 0.2 (precision)	200	—	<50	<5000
HTC Vive Pro Eye	Integrated	0.5−1.1 (accuracy) 0.4 (precision) Schuetz and Fiehler (2022)	120	—	<50	∼1000
Pupil Labs Neon Pico 4	Integrated	1 (accuracy) 0.08 (precision)	200	—	<20	<15000
Varjo XR-4	Integrated	1 (accuracy) 0.2 (precision)	200	—	<30	<10000

#### 4.3.1 Tower-mounted eye trackers

Tower-mounted eye trackers, such as the *EyeLink 1000 Plus* and *Tobii Pro Spectrum*, are high-precision systems designed for controlled environments. These devices use high-resolution cameras and infrared illumination to capture detailed eye movements, often requiring head stabilization via a chinrest or bite bar to minimize external interference. They achieve superior spatial accuracy and sampling rates up to 2000 Hz, enabling the study of fine fixational eye movements, saccades, and rapid phases of nystagmus, critical for neurophysiological and vision research (Nyström et al., 2021). These systems capture raw monocular eye position data, including pupil center coordinates and corneal reflection positions for each eye, at high temporal resolution. Algorithms process this into cyclopean eye position, averaging binocular data to estimate gaze direction, or compute gaze coordinates mapped to a stimulus plane via calibration (Holmqvist and Andersson, 2017). Access to raw monocular data, crucial for detailed oculomotor analyses like microsaccades, often requires premium software licenses—*e.g.*, EyeLink Data Viewer, Tobii Pro Lab—while processed gaze data is standard (Nyström et al., 2016).

Traditionally, tower-mounted eye trackers, such as the EyeLink 1000 Plus, have employed classic computer vision algorithms for pupil detection, corneal reflection tracking, and gaze estimation, avoiding reliance on artificial intelligence (AI) (Holmqvist et al., 2011). These deterministic approaches, utilizing techniques like thresholding and geometric modeling, are tailored for highly controlled laboratory settings where precision, speed, and reliability are paramount, particularly at sampling rates up to 2000 Hz (Holmqvist et al., 2011). Head stabilization via chinrests and consistent lighting ensure robust performance without AI (Gibaldi et al., 2017). Recent advancements in eye-tracking technology, however, highlight growing exploration of AI and machine learning, particularly in less controlled environments (Klaib et al., 2021; Tonsen et al., 2016). Although not yet a standard feature in high-precision tower-mounted systems like the *EyeLink 1000 Plus*, AI algorithms demonstrate potential for improving pupil detection under challenging conditions, such as blinks, minor head movements, or reflections from glasses, and for enabling adaptive calibration and predictive gaze estimation to mitigate brief tracking losses (Klaib et al., 2021; Fuhl et al., 2017). Deep learning models are integrated cautiously in high-precision systems due to potential latency, which can undermine the millisecond-level accuracy required for experimental research (Andersson et al., 2017).

A few key considerations are worth noting. First, while these eye trackers can achieve high sampling rates and spatial accuracy under head-constrained conditions, studies have identified certain limitations in pupil-based systems when recording the dynamics of saccadic eye movements (Nyström et al., 2016). Indeed, during saccadic movements, the rapid accelerations exerted by the eye muscles induce significant forces on the eyeball, causing changes in pupil size and center position. These changes can distort the velocity profiles of saccades recorded by pupil-based trackers, and thus variations in saccadic measurements across participants and experimental conditions should be interpreted with caution. For experimental paradigms that require precise measurement of oculomotor dynamics, alternative tracking technologies discussed in Section 4.1 may be more suitable, although these require highly controlled environments.

Second, some tower-mounted systems can be adapted for head-free eye tracking, which is advantageous in studies where head stabilization is impractical but high accuracy is still required—*e.g.*, in developmental research involving infants (Hessels and Hooge, 2019). This head-free adaptation may lead to a slight reduction in sampling frequency and accuracy, thereby blurring the line between tower-mounted and remote eye trackers, despite technical and price differences between the two categories as discussed in subsequent sections.

#### 4.3.2 Remote eye-trackers

Remote eye trackers, such as the *Tobii Pro Spark*, *GazePoint GP3*, and *EyeLogic LogicOne*, are compact systems positioned below a stimulus display, typically a computer screen, using infrared light and cameras for gaze tracking. With sampling rates of 60–250 Hz, they prioritize ease of use and participant comfort, eliminating the need for head stabilization, thereby allowing participants to engage in a natural and unobstructed viewing experience. This less intrusive setup is advantageous for usability testing, a range of human behavior and visual psychology studies, as well as screen-based market research (Niehorster et al., 2018). These systems collect raw monocular eye position data, such as pupil center and corneal reflection coordinates, but typically output processed gaze coordinates mapped to a screen-based calibration plane using polynomial regression or neural network models (Morimoto and Mimica, 2005). Raw monocular data, useful for studying binocular coordination, is often accessible only through premium subscriptions—*e.g.*, Tobii Pro SDK, GazePoint SDK—as processed gaze data is the default (Niehorster et al., 2018).

These systems typically allow for some degree of head movement accommodation, but excessive motion can result in data gaps, inaccuracies, and artifacts. Specifically, remote eye-tracking systems feature a constrained functional area, known as a *head box* and often restrict gaze tracking to a designated *calibration plane*, usually the computer screen. If a participant moves outside the head box or looks beyond the calibration plane, tracking may pause temporarily. High-quality systems can rapidly reacquire eye movements with minimal data loss when the participant’s gaze returns to the calibration plane. However, substantial shifts in distance from the screen can necessitate recalibration, and changes in vergence—eye convergence—may introduce additional error in tracking data.

Artificial intelligence (AI) is transforming the capabilities of remote eye trackers, enhancing their robustness, accuracy, and usability across diverse environments. Traditionally, remote eye trackers, which rely on infrared illumination and corneal reflection without physical contact, required controlled lighting, stable head positioning, and careful calibration (Holmqvist and Andersson, 2017). Early systems mainly used rule-based image processing methods for pupil and glint detection, but these approaches struggled with real-world challenges like head movements, glasses reflections, partial occlusions, and changing ambient lighting (Santini et al., 2018). To address these issues, AI-driven methods, particularly deep learning models, are now widely adopted for tasks such as real-time pupil detection, gaze estimation, and dynamic head pose compensation (Santini et al., 2018). Convolutional neural networks (CNNs) enable robust eye feature extraction even under suboptimal imaging conditions, while machine learning models trained on large datasets predict gaze direction more accurately across a wide range of head positions, facial geometries, and lighting environments (Zhang et al., 2017; Fuhl et al., 2017; Ansari et al., 2023). Deep learning also supports user-adaptive gaze estimation, fine-tuning calibration based on individual anatomical or behavioral differences (Byrne et al., 2025), thus facilitating *“low-calibration”* operation (Liu et al., 2018).

When selecting an eye-tracker, researchers often weigh specifications such as spatial accuracy, precision, and head box dimensions. Manufacturers typically present these metrics as representative for any participant within the head box, though they may overlook the limitations of non-ideal head positioning. However, empirical studies (Niehorster et al., 2018) have shown that these specifications are most reliable only when participants closely adhere to instructions and maintain optimal positioning. When participants deviate from these optimal conditions (Hessels et al., 2015b), or when recording from challenging groups—like infants (Hessels et al., 2015a) — both accuracy and precision can degrade significantly, even if the eyes remain within the head box. This can lead to considerable data loss and reduced data quality, with important implications for subsequent data analysis and interpretation.

#### 4.3.3 Wearable eye-trackers

Wearable eye tracking systems, often referred to as head-mounted or mounted eye tracking systems, typically consist of lightweight and ergonomically designed eyewear or headbands, such as *Pupil Labs Neon* and *Tobii Pro Glasses 2/3*. These systems generally incorporate one or more cameras positioned within the visual field of one or both eyes, alongside an additional camera that captures the surrounding scene or field of view. In head-mounted configurations, gaze tracking is performed relative to the entire field of view, making these systems particularly well-suited for real-world experimental settings. Wearable eye trackers are employed across a diverse range of research applications, including decision-making studies in marketing (Gidlöf et al., 2017), analysis of viewing behaviors among various professional groups (McIntyre et al., 2019), investigations into shared manipulation in human–robot interaction (Aronson et al., 2018), and examinations of social interactions among adults Macdonald and Tatler (2018). They capture raw monocular eye position data and output gaze coordinates (MacInnes et al., 2018b). Pupil Labs provides raw data openly via Pupil Capture, but Tobii restricts unprocessed data and advanced mapping tools—see below—to premium subscriptions—*e.g.*, Tobii Pro Lab Glasses Edition) (Macdonald and Tatler, 2018).

The miniaturization and portability of wearable eye-tracking systems, while advantageous for real-world applications, come with trade-offs in terms of performance. For instance, while high-end fixed laboratory eye trackers can record eye positions at frequencies up to 2000 Hz, modern wearable eye-tracking glasses typically have lower recording capacities, with most devices operating within the 50−100 Hz range, and the upper limit rarely exceeding 250 Hz. This reduced sampling rate limits the utility of wearable eye trackers in studies requiring high temporal precision, such as investigations into fine fixational eye movements, or the recordings of saccades and fast phase of the VOR and OKN. For example, exploring the non-linearity of saccade trajectories or examining microsaccades is generally considered unfeasible with eye-tracking systems that sample below 250 Hz, as these phenomena require higher-frequency data capture for accurate analysis (Martinez-Conde et al., 2009). Additionally, maintaining an unobstructed view of the eyes is essential for accurate tracking, making it difficult to capture peripheral eye movements. This can lead to a decrease in tracking accuracy, particularly in dynamic or less controlled environments.

Artificial intelligence (AI) has become integral to modern wearable eye trackers, significantly enhancing their flexibility, accuracy, and usability in real-world environments. Unlike fixed, laboratory-based systems, wearable devices must contend with constant head movements, changing lighting conditions, partial occlusions from eyelids or glasses, and variations in individual anatomy (Holmqvist and Andersson, 2017; Tonsen et al., 2016). To address these challenges, AI-driven algorithms, particularly deep learning models, are employed for real-time pupil detection, gaze estimation, and robust calibration without extensive manual setup (Klaib et al., 2021; Yiu et al., 2019). For instance, the DeepVOG framework utilizes fully convolutional neural networks for pupil segmentation and gaze estimation, demonstrating robust performance across multiple datasets (Yiu et al., 2019). Similarly, YOLO-based models have been applied for accurate pupil center detection in visible-light conditions (Ou et al., 2021).

Neural network-based approaches adeptly handle issues such as reflections from glasses, variable illumination, and motion artifacts, ensuring reliable gaze tracking during dynamic activities like walking, driving, or sports analysis (Chaudhary et al. (2019); Tonsen et al. (2017). Trained on diverse datasets, these models adapt to the complex visual conditions typically encountered outside controlled environments (Garbin et al., 2019; Tonsen et al., 2016). In settings where lighting is consistent and computational resources are limited, simpler techniques like edge detection can complement AI-based tracking for greater efficiency (Holmqvist and Andersson, 2017). Wearable eye trackers require compact AI models optimized for lightweight cameras and minimal infrared illumination, with fast, adaptive calibration to maintain tracking accuracy on the move (Fuhl et al., 2017; Tonsen et al., 2017). Recent advancements, such as lightweight convolutional neural networks, have improved real-time pupil detection for mobile eye tracking, although challenges related to model compression and energy efficiency remain critical for deploying deep learning on embedded systems (Marvasti-Zadeh et al., 2021).

Furthermore, wearable eye-tracking devices generate gaze data based on a coordinate system defined by both the wearable tracker and the recorded scene video, rather than being anchored to fixed objects in the observer’s visual field. When the participant’s head and body movements are unconstrained, a disassociation between eye movements and gaze shifts can occur. This creates a challenge, especially as many recent studies aim to investigate gaze dynamics in naturalistic environments, where it is crucial to translate gaze data into a consistent, fixed frame of reference. To overcome this challenge, manual mapping of gaze coordinates to a fixed reference frame can be time-consuming and typically feasible only for short recordings. Several automated solutions have been developed to address this issue. For example, certain systems use markers to delineate a consistent tracking area, such as a section of the visual field or a screen, to maintain reference during data collection. In addition, techniques exist to map eye-tracking data onto a more static representation of the scene. *Tobii*, for instance, offers the *Real World Mapping* (RWM) solution, while *Pupil Labs* has developed the *Reference Image Mapping* (RIM) module, both of which enable the conversion of dynamic gaze data into a fixed spatial framework. It should also be mentioned that open-source pipelines for gaze mapping can be found in the literature (MacInnes et al., 2018b; MacInnes et al., 2018a).

#### 4.3.4 Integrated eye-trackers

This category includes eye-tracking devices embedded within a variety of technological systems. Notable examples include aiming systems in eye surgery technologies, other medical devices, and eye-tracking systems integrated into vehicle dashboards. Recent advances in virtual and augmented reality (VR/AR) technologies have significantly enhanced the quality and accessibility of integrated eye-tracking systems, facilitating their widespread adoption in research environments. VR provides a fully immersive experience, while AR augments the user’s real-world environment by overlaying digital elements onto live visual input—more detailed information on this can be found in the review by Clay et al. (2019). These systems collect monocular eye position data (pupil coordinates, corneal reflections) but prioritize processed 3D gaze coordinates or vergence-based depth in virtual environments, using calibration to map gaze to stimuli (Duchowski et al., 2000). Raw monocular data, valuable for custom research—*e.g.*, vergence studies—often requires premium licenses—*e.g.*, Tobii’s XR SDK—while processed gaze data is standard for applications like foveated rendering (Lang et al., 2018).

Eye tracking in VR/AR is a relatively recent innovation, first emerging in the literature around the turn of the century (Duchowski et al., 2000), and offers substantial potential for advancing research on human perception and behavior. In contrast to traditional eye-tracking methods, VR/AR enables the creation of controlled experimental settings while granting participants the freedom to move within a relatively naturalistic environment (Clay et al., 2019; Drewes et al., 2021). Participants can explore their surroundings by moving their heads, and the precise spatial relationship between stimuli and the participant’s gaze can be tracked concurrently with head movements.

Similarly to wearable and remote eye trackers, artificial intelligence (AI) is playing a critical role in advancing AR/VR eye tracking systems, making gaze estimation faster, more accurate, and more adaptive to immersive environments. Deep learning algorithms enable personalized calibration by modeling individual anatomical variations, such as corneal curvature and interpupillary distance, reducing setup time and improving user comfort (Liu et al., 2018). AI also supports predictive gaze models, allowing systems to anticipate eye movements for foveated rendering, where high-resolution graphics are focused only where the user is looking, enhancing performance and realism (Arabadzhiyska et al., 2017; Patney et al., 2016).

Eye tracking in virtual and augmented reality enables diverse applications across multiple domains, including education and training (Lang et al., 2018), clinical diagnostics (Miao et al., 2020), and marketing and consumer research (Meißner et al., 2019), among others. Gaze-based pointing and target selection introduce intuitive multimodal interaction methods (Jacob and Stellmach, 2016; Majaranta and Bulling, 2014; Plopski et al., 2022), while awareness of a user’s gaze direction enables novel approaches for navigation and subtle manipulation of virtual environments (Langbehn et al., 2018; Marwecki et al., 2019). The broad applications of VR-based eye tracking span numerous fields. For a comprehensive overview see the recent work by Adhanom et al. (2023).

In terms of hardware, key manufacturers in the field include *Tobii* and *Pupil Labs*. *Tobii* integrates its eye-tracking technology directly into VR headsets such as the *HTC Vive Pro Eye*, while *Pupil Labs* provides modular add-ons that can be attached to commercial headsets without built-in eye tracking, such as the *Pico 4*. Due to the relatively recent development of eye tracking in VR, assessing the quality of specific hardware can be challenging at times. Similar to *traditional* eye-tracking systems, there is a growing demand for standardization of data quality reporting in VR. Notably, Adhanom et al. (2020) introduced an open source package to measure gaze precision and accuracy within the Unity rendering engine.

## 5 Discussion

Much of our understanding of the role of various oculomotor control circuits in complex tasks is derived from extrapolating results obtained in simple laboratory experiments. The fundamental characteristics of eye movements under these controlled conditions are well-documented in the eye-tracking literature, and such environments allow for reproducibility and comparison across studies. Laboratory settings offer several advantages, particularly in terms of stimulus design. Visual stimuli can be carefully constructed based on known physiological parameters, including those of theoretical relevance to the experiment. The tasks are typically designed to be simple, with the key experimental parameters clearly defined, allowing the stimulus to contain most of the relevant information needed for the task. As a result, behaviors can be easily quantified in parametric terms, such as reaction times to stimulus presentation, and the task can be explicitly instructed and its difficulty level precisely controlled. This creates a situation akin to a toy model, where nearly all variables are controlled, enabling a detailed understanding of the processes involved.

However, this approach has notable limitations. The controlled, simplified nature of laboratory experiments creates nonphysiological scenarios that may not accurately reflect real-world conditions. Although laboratory research can provide valuable information on the neurophysiological mechanisms underlying basic oculomotor control, it may have limited applicability to the characterization of more complex behavioral processes that occur outside of these controlled settings. As a result, while such studies are invaluable for understanding the fundamental building blocks of eye movement control, they may not fully characterize more complex behavioral processes expressed in more naturalistic, real-world contexts.

In this respect, research carried out under less controlled conditions is interesting from two points of view. Firstly, as discussed in Section 3, a comprehensive understanding of the visual system requires an analysis that integrates not only the visual processes themselves but also their interaction with other body segments and functions, such as head and upper limb movements. Secondly, studying visual function in more natural contexts allows for a more ecologically valid analysis of integrative visual behavior, something that is typically not achievable with brain imaging techniques like fMRI or basic neurophysiological methods. Therefore, it may appear paradoxical that the study of visual behavior, particularly when integrated with physical or behavioral tasks, has been so limited in scenarios where the participant’s head movement is not restricted.

While naturalistic research is essential for understanding which visual strategies, enabled by the flexibility of the human oculomotor system, are employed in real-world tasks, and what role eye movements play in these strategies, extrapolating findings from laboratory experiments to natural settings is not always as straightforward as it might seem. One of the key challenges in naturalistic environments is designing and parameterizing stimuli in ways that reveal essential behavioral components. Furthermore, characterizing natural eye movements remains complex as they are often the result of the interaction between multiple neural pathways. For example, vestibular inputs combine with visual information to accomplish a motor task, making it difficult, if not impossible, to isolate the contribution of each system to a given movement. This suggests that the modular approach to oculomotor control, which posits that each eye movement is governed by a distinct neural circuit, is insufficient to adequately describe natural eye movements (Steinman et al., 1990).

Interestingly, this issue reflects the broader *real-world or the lab* dilemma in cognitive science and psychology, as outlined by Hammond and Stewart (2001). Specifically, the artificial nature of experimental environments may differ from real-world contexts in ways that make the results less relevant to the phenomena that researchers aim to explain. The field has experienced what has been referred to as an *ecological validity crisis* (Aanstoos, 1991), many authors have noted that *“what is missing is an independent, objective, and operational definition of the concept of ecological validity”* (Lewkowicz, 2001) which could jointly cover the nature of the stimuli, the nature of the task and the nature of the research context. These concerns are central to recent discussions on the limitations of ecological validity in eye-tracking research (Holleman et al., 2020). Although these questions remain unresolved, they encourage researchers to clearly specify and describe the environmental contexts they study, which helps uncover contextual and general principles of behavioral and physiological mechanisms.

To address the limitations described above, two main approaches have emerged in the literature. The first approach involves studying gaze behavior under laboratory conditions. This approach aims to isolate the contributions of different systems involved in eye movement control, indirectly returning to a modular perspective of the oculomotor system. Typically, participants are asked to direct their gaze to a specific location when a stimulus appears, and a set of metrics is used to characterize the coordination and interdependence of the subsystems involved in gaze control. However, this approach is not without limitations, as it relies on reductionist paradigms that may not accurately reflect real-world behavior. The second approach focuses on recording natural eye movement behavior, acknowledging that determining the precise neural sources of each movement is unlikely. This approach shifts the focus to the analysis of eye movements from a task-oriented perspective, a relatively new area in eye tracking research (Lappi, 2016). Ultimately, there exists a continuum between control and realism, and it is up to each researcher to balance these two perspectives in their experimental design. The challenge lies in finding an appropriate balance between simplifying the system for robust experimental outcomes and capturing the rich complexity of real-world behavior while navigating the potential limitations of both approaches.
